# Functionalized Graphene Oxide for Chemotherapeutic Drug Delivery and Cancer Treatment: A Promising Material in Nanomedicine

**DOI:** 10.3390/ijms21176280

**Published:** 2020-08-30

**Authors:** Horrick Sharma, Somrita Mondal

**Affiliations:** Department of Pharmaceutical Sciences, College of Pharmacy, Southwestern Oklahoma State University, Weatherford, OK 73096, USA; somrita.mondal@swosu.edu

**Keywords:** nanoparticles, graphene oxide, GO–metal nanoparticles, targeted drug delivery system, cancer therapy, nanomedicine

## Abstract

The usage of nanomaterials for cancer treatment has been a popular research focus over the past decade. Nanomaterials, including polymeric nanomaterials, metal nanoparticles, semiconductor quantum dots, and carbon-based nanomaterials such as graphene oxide (GO), have been used for cancer cell imaging, chemotherapeutic drug targeting, chemotherapy, photothermal therapy, and photodynamic therapy. In this review, we discuss the concept of targeted nanoparticles in cancer therapy and summarize the in vivo biocompatibility of graphene-based nanomaterials. Specifically, we discuss in detail the chemistry and properties of GO and provide a comprehensive review of functionalized GO and GO–metal nanoparticle composites in nanomedicine involving anticancer drug delivery and cancer treatment.

## 1. Introduction

**Figure 1:**
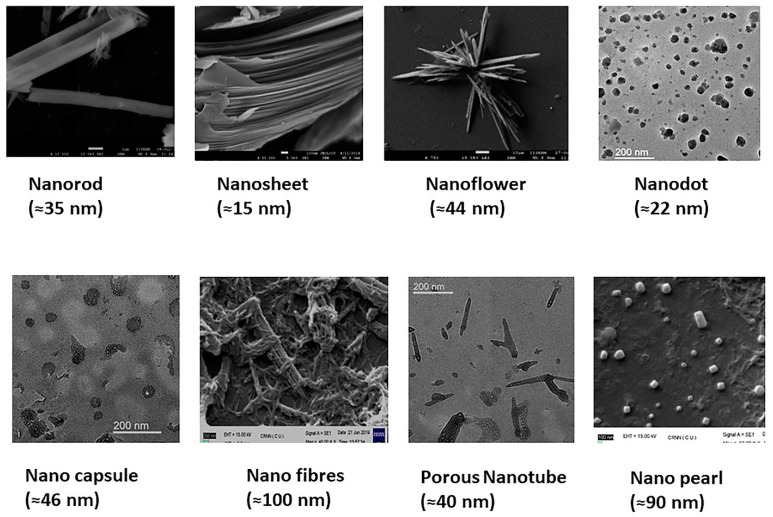
Nanomaterials of different shapes and sizes.

Cancer is the second-leading cause of mortality, with over 8 million deaths worldwide [1]. Chemotherapy remains one of the most common treatment modalities for cancer. Conventional drug delivery systems and treatment approaches have several limitations, including low aqueous solubility of small molecules, rapid metabolism and elimination of drugs, failure to attain the desired target site concentration, multi-drug resistance, and non-specific cytotoxicity. To address the above limitations, the use of nanomaterials in cancer therapy has led to some cutting-edge research during the last few years [2,3,4,5,6,7]. Nanomaterials are chemical substances with dimensions between 1 and 100 nm. Nanoparticles can be composed of either organic, inorganic, or hybrid materials and can take the shape of a tube, wires, ribbons, needle, sphere, capsule, rod, disc, dots, plate, or a cube (Figure 1) [8]. On the basis of the overall shape, nanostructured materials can be zero-dimensional, 1D, 2D, or 3D. They are mostly colloidal systems, made of metals, metal oxides, semiconductors, carbon, and polymers [9]. Nanoparticles’ surfaces can have different properties on the basis of the surface charge, size, hydrophobic or hydrophilic functional groups, presence of coating materials, and targeting ligands [10].

Organic nanoparticles such as liposomes, solid lipid nanoparticles, dendrimers, polymeric nanoparticles, hydrogels, and polymeric micelles are widely used for drug, gene, or siRNA delivery, cancer therapy, and diagnostics [11]. Carbon-based nanomaterials include fullerenes [12], carbon nanotubes [13], carbon dots [14], carbon nanofibers [15], graphene [16], graphene oxide (GO) [17], reduced graphene oxide (rGO) [18], and graphene quantum dots [19]. Inorganic nanomaterials include metal or metal oxide nanoparticles, including iron oxide (FeO) nanoparticles [20,21], zinc oxide (ZnO) nanoparticles [22,23], silver nanoparticles (AgNPs) [24,25], gold nanoparticles (AuNPs) [26,27], mesoporous silica nanoparticles (SiNPs) [28], and group II–VI core and core-shell quantum dots (CdSe, CdS, ZnSe, ZnS, etc.) [29,30,31,32,33,34,35,36,37,38,39,40], among others. Inorganic nanoparticles have different physical and chemical properties such as inertness, stability, and ease of functionalization. Metal, semiconductor, and graphene-based nanoparticles have excellent optical and electrochemical properties such as fluorescence, surface plasmon resonance, and Raman enhancement that enable their use as sensors and transducing devices. The paramagnetic and superparamagnetic inorganic nanoparticles, for example, FeO nanoparticles, have found molecular imaging applications such as computed tomography, magnetic resonance imaging, or positron emission tomography as an image contrast agent [41]. Inorganic nanoparticles have characteristic near-infrared (NIR) absorption or emission for luminescence imaging. Since the NIR light can penetrate deeper into and out of biological tissues when compared with the UV, visible, or far IR light, nanomaterials, which can absorb in NIR region, are more efficient in converting optical energy into thermal energy and have found clinical applications in photothermal therapy (PTT) [42,43,44].

**Figure 2:**
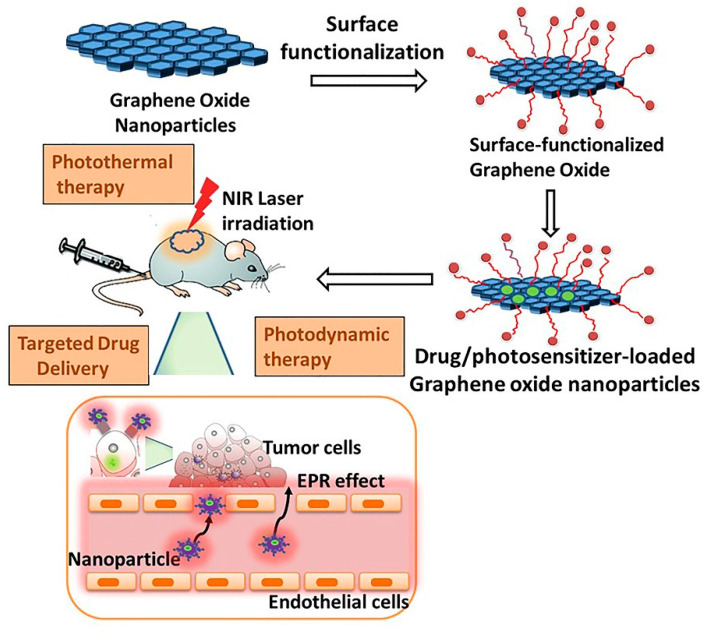
Surface functionalization of graphene oxide (GO) nanoparticles (NPs) and loading of drug and photosensitizer on the surface-modified GO–metal NPs. Finally, the application of GO nanocomposites for targeted drug delivery and in vivo photodynamic therapy using the near-infrared (NIR) laser irradiation is shown.

In this review, we provide an update on recent developments in the biomedical applications of functionalized GO nanomaterials and their composites, focusing on their use as nanocarriers for cancer-targeted drug delivery and cancer therapy (Figure 2). Firstly, we discuss the concept of nanoparticle-mediated tumor targeting. Next, we review the chemistry and critical properties of graphene oxide and their functionalization for biomedical applications. We elaborate in detail on the application of surface-modified GO composites for anticancer therapy, cancer cell imaging, and nanocarrier for cancer therapeutics. Finally, we briefly summarize in vivo biocompatibility of graphene-based nanomaterials (GBNs) and current limitations and challenges in the use of GBNs in biomedical applications.

## 2. Nanoparticle-Mediated Tumor Targeting

Conventional chemotherapeutics lack specificity for tumor cells and are associated with dose-limiting cytotoxicity. Localizing the chemotherapeutic drug at the tumor site and targeted drug delivery into the tumor cell can reduce the side effects of chemotherapy. Passive targeting by nanoparticles is one approach that takes advantage of the tumor’s unique pathophysiological features to deliver therapeutic agents from the nanocarriers at the tumor site. Unlike the normal vasculature, which is impermeable to molecules of size >2–4 nm, tumors have leaky vasculature that is highly disorganized and dilated with a higher number of pores. Moreover, cancer has compromised lymphatic drainage, further facilitating the stagnation of nanoparticles within the tumor environment. Over the past decade, significant research has been carried out on building nanosized carriers for targeted delivery of anticancer agents [45,46]. Nanomaterials have a higher surface-to-volume ratio and exhibit enhanced permeability and retention (EPR) effect, ligand-mediated active tumor targeting, and controlled and triggered drug release [47]. Nanoparticle-based drug delivery systems improve the solubilization of poorly soluble chemotherapeutics and prolong their circulation in the vasculature. Their unique optical, magnetic, and electrical properties have found a wide range of commercial applications in cancer diagnostics and therapy [48,49,50,51,52,53].

Nanoparticles can be administered by various routes, including oral, parenteral, and pulmonary inhalation depending upon the disease state, the drug target, patient compliance, and the material and surface properties of nanomaterials [54]. Among these, oral and intravenous administration are the two most common routes of administration of NPs. Concerning the administration of nanoparticles via inhalation, the barriers to drug delivery include clearance in the upper airway by ciliated epithelial cells and in the lower airway by lung-associated macrophages [55]. The oral administration of a dug through encapsulating NPs, in general, improves drug bioavailability because of enhanced permeation and absorption of nanomaterials. Orally administered NPs encapsulate and protect the acid-sensitive or peptidomimetic drug against chemical and metabolic degradation in the gastrointestinal tract, and enable controlled and systemic release of drugs. Nanoparticle formulation further improves the delivery of poorly water-soluble drugs, mediates transcytosis across the tight intestinal barrier, reduces transporter-mediated efflux of drugs, and enables intracellular and transcellular delivery of large macromolecules [54,55]. Nanoparticle therapeutics also reduce the hepatic first-pass effect of oral medications. The intravenous administration is the other common route of administration of NPs that provides immediate effect, reduces the first-pass, and avoid renal clearance of NPs sized >15 nm. However, one of the most significant limitations of nanoparticle therapeutics is clearance by the reticuloendothelial system (RES) [56]. After absorption through oral administration or following intravenous administration, NPs undergo opsonization and sequestration in the systemic circulation. Non-functionalized NPs are cleared from the bloodstream by the mononuclear phagocyte system of the liver or the spleen, that comprises the RES, through phagocytosis or pinocytosis. Since most NPs are between 50 and 200 nm in size, they are mostly cleared by phagocytosis [57]. Upon contact with blood, based on their surface characteristics, size, and morphology, NPs attract different opsonins and other plasma proteins that become attached to its surface, resulting in the formation of a protein corona. After protein corona formation, NPs get attached to specific receptors on the surface of phagocytes, which internalize the NPs, transport them to phagosomes, which then undergo fusion with lysosomes, leading to their degradation and finally excretion by urinary clearance.

**Figure 3:**
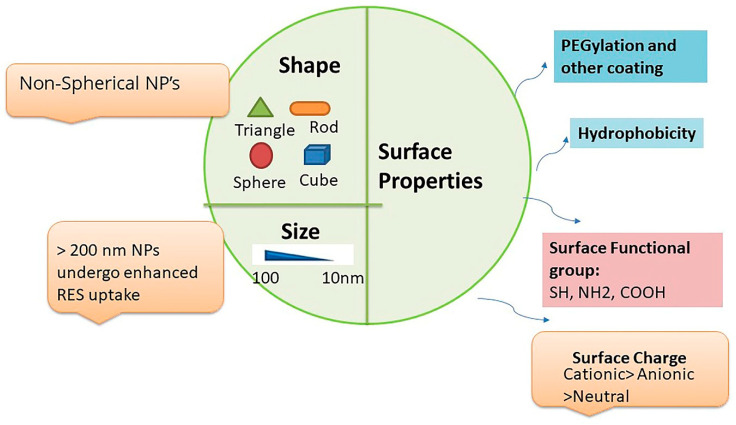
Physicochemical and surface properties of nanoparticles (NPs) that affect protein formation and reticuloendothelial system (RES) uptake. Particle size, shape, surface charge, lipophilicity, functional groups and polyethylene glycol (PEG)ylation influence the opsonization and RES uptake of NPs. Non-spherical shaped NPs of ≈100 nm undergo less RES uptake than spherical and larger-sized NPs. PEGylation, hydrophobicity, and slightly anionic or neutral zwitter ionic particles hinder protein corona formation and undergo less RES accumulation.

Prolongation of NPs blood circulation would require lower RES accumulation, which is governed by physico-chemical factors of NPs such as size, shape, charge, and surface properties (Figure 3) [58]. Size is one of the most critical factors that control the circulatory residence time and uptake of NPs by the RES. It has been shown that NPs sized >40 nm are cleared slowly (6 months), while NPs <15 nm are cleared within 24 h [55]. Notably, small-sized particles <5 nm after undergoing rapid uptake in the liver and spleen are also rapidly removed by kidneys. Further, NPs sized >200 nm undergo higher accumulation in organs such as the liver and spleen [57]. Overall, nanoparticles with a particle size of ≈100 nm tend to have prolonged circulating half-lives. Surface properties of NPs, such as charge, also influence their RES uptake. Surface charge affects protein adsorption of NPs, which in turn change their opsonization and sequestration properties. Positively charged NPs undergo higher uptake by RES and are rapidly cleared from circulation to a greater extent than the slightly negative or neutral (zwitter ion) charged nanoparticles. It is particularly true for the small-sized neutral vesicles, which are not efficiently coated with the opsonins and are poorly recognized by phagocytic cells. However, even for neutral and anionic NPs, their clearance rates generally increase with increasing nanoparticle size. In addition to the size and surface charges, nanoparticle shape significantly affects their transport and diffusion properties. Spherically shaped particles are reported to move more swiftly with blood when compared with non-spherical particles that shows tumbling and rolling dynamics in the vasculature. The non-spherical NPs show higher accumulation in the liver, spleen, and kidneys than their spherical counterparts. The non-spherical NPs may also exhibit long-term circulation in the blood and could be more effective than spherical nanoparticles for drug delivery. Another crucial strategy to avoid RES uptake is the functionalization of NPs with polyethylene glycol (PEG), which contains hydrophilic ethylene glycol units that hinder protein adsorption. Further, “stealth” particles are being explored to avoid RES clearance and prolong the circulation time of NPs in blood [59].

Circulating NPs that escape RES surveillance exit tumor blood vessels by diffusion and conviction across the microvascular tumor wall and enter the tumor interstitium. Impaired lymphatic drainage in neoplastic tissues, along with leaky tumor blood vessels, lead to the EPR effect, resulting in higher accumulation of the drug in cancerous interstitium than in plasma and other tissues [60,61]. For nanoparticles to extravasate the vasculature and circumvent renal and liver clearance, they should preferably possess neutral or negative charge and be within 10–100 nm in size [62,63,64].

**Figure 4:**
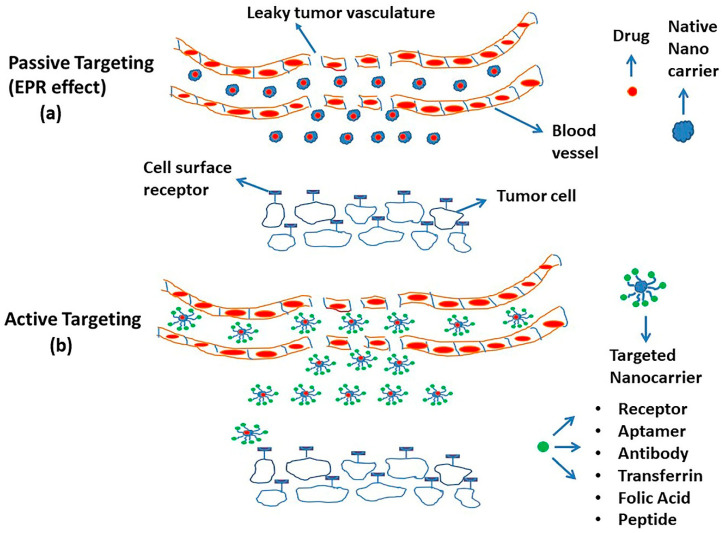
The figure shows passive (enhanced permeability and retention (EPR) effect) (**a**) and active tumor targeting (**b**) of nanoparticles (NPs) for targeted drug delivery. Tumors have disorganized and leaky vasculature with a higher number of pores facilitating the movement of NPs from the vasculature and their accumulation in the tumor environment. Moreover, active targeting makes use of targeting ligands that are attached to NPs and bind specifically to cell surface receptors of tumor cells.

Despite numerous successful applications in preclinical animal models, only a few passive targeting-based drug delivery systems are approved for clinical use [65]. The Food and Drug Administration (FDA)-approved drug delivery systems for cancer therapy include PEGylated liposomal doxorubicin (Doxil), PEGylated liposomal daunorubicin (DaunoXome), albumin-bound paclitaxel NP (Abraxane), liposomal daunorubicin and cytarabine combination (Vyxeos), liposomal irinotecan (Onivyde), liposomal cytarabine (DepoCyt), and liposomal vincristine (Marqibo) [66,67]. Active targeting is an attractive approach that, in addition to EPR, makes use of surface-functionalized nanoparticles [61,68]. Here, the uptake of drug-loaded NPs results from a precise interaction of the targeting moieties on NPs and surface receptors on tumor cells. One unique example of active targeting is vascular targeting, in which the NPs are designed to interact with the vascular endothelial growth factor (VEGF) receptors localized on the vascular endothelial cells within the tumor vasculature [69]. While some nanoparticle formulations are made to dissociate in the tumor interstitium, most NPs are designed to enter the tumor cell intact and release the encapsulated drug intracellularly. Examples of targeting moieties attached to the surfaces of NPs for active targeting include antibodies, peptides, proteins, aptamers, and small molecules [70,71]. These ligands bind specifically to the antigen, for example, CD19 antigen and cell-surface receptors, including human epidermal growth factor receptor 2 (HER2), epidermal growth factor receptor (EGFR), transferrin, sigma receptors, VEGF receptors, folate receptors, and glycoprotein receptors expressed on the tumor cell surface [72]. The schematic representation of active and passive targeting by NPs is illustrated in Figure 4. Another limitation of nanocarriers involves insufficient release specifically at the tumor site. Enhanced specific drug release could be achieved through stimuli-responsive NPs sensitive to changes in the tumor microenvironment and the tumor cell, e.g., hypoxia; low intracellular pH or increased concentration of enzyme proteases, peptidases, and glutathione; or physical stimuli such as temperature, acoustics, and light. In addition to passive and active targeting, the development of such stimuli-responsive “smart” nanoparticles may overcome barriers to tumor heterogeneity and provide enhanced and selective uptake and release of chemotherapeutics at the target tumor site [73,74,75,76].

## 3. Graphene-Based Nanomaterials

**Figure 5:**
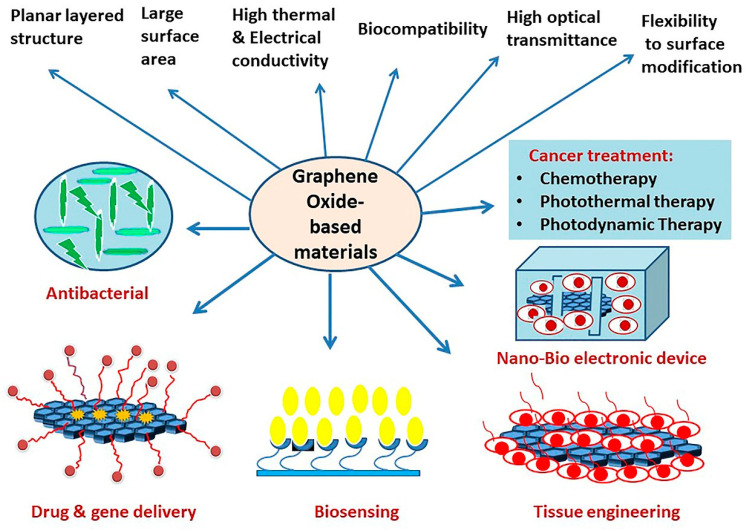
The figure illustrates important properties of graphene oxide (GO) and biomedical applications of GO-based nanomaterials and nanocomposites.

Graphene has been a matter of intense research since its first arrival in 2004, owing to its exclusive structure and properties such as optimal thermal (≈5000 W/mK) and electrical conductivity (6000 S/cm), high optical transparency (up to 97%), high Young’s modulus (≈1 Tpa), ambipolar field effect with excellent charge carrier mobility (ranging from ≈200,000 to ≈500,000 cm^2^/Vs), huge available surface area (2630 m^2^/g), breaking strength (130 GPa), mechanical stiffness (1060 GPa), unique surface functionalization capability, and biocompatibility [77,78,79,80]. The zero band gap of graphene, sharp edges, the need for doping, and its hydrophobicity and poor solubility in most solvents require functionalization of graphene for its wide array of applications [81,82]. The family of graphene-based nanoparticles (GBNs) includes GO; rGO; graphene quantum dots; and graphene nanocomposites with inorganic, polymer, and organic nanomaterials [83,84]. GBNs and their functionalized composites have garnered more interest due to their amenable chemical, mechanical, electrical, thermal, and optical properties. Because of several favorable features (Figure 5), GO and their composites have found potential applications in optics, electronics, nanocatalysis, and nanomedicine, including biosensing, targeted drug delivery system, cellular imaging probes, tissue engineering, antibacterial application, PTT, and photodynamic therapy (PDT) [85,86,87,88,89,90,91,92,93,94,95,96].

## 4. Graphene Oxide Nanoparticles: Synthesis, Unique Properties, and Biomedical Applications of GO-Based Nanomaterials and Nanocomposites

**Figure 6:**
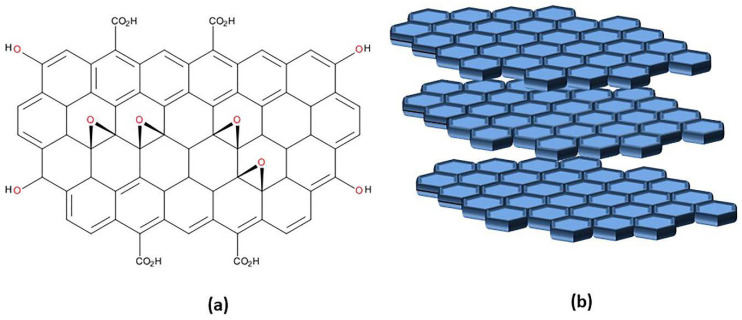
Chemical structure of graphene oxide (GO) (**a**) and multilayered planar structural arrangement of GO (**b**).

The structure of graphene is a monolayer of two-dimensional (2D) one atom thick planar, sp^2^-hybridized carbon arranged in six atom rings [97]. Graphene can favorably stack and exists as bi-layer or multi-layer sheets. X-ray diffraction (XRD) shows that graphene forms a honeycomb crystal lattice structure. Graphene is synthesized by two common methods: the top-down approach, which is based on reducing or breaking down larger-scale material to nanoscale elements, and the bottom-up approach, where graphene is built from smaller carbon precursors [98]. In a top-down method, graphene is produced through mechanical exfoliation of graphite, liquid-phase exfoliation, ball milling, and oxidation of graphite followed by reduction of graphene oxide. Bottom-up approaches include chemical vapor deposition, epitaxial growth, unzipping of carbon nanotubes, and organic synthesis. GO is an oxidized and more water-soluble derivative of graphene. It is synthesized from graphene using strong oxidizing agents by more popular Hummer’s (or its modified) method or through Brodie’s or Staudenmaier’s process. Hummer’s method uses potassium permanganate (KMNO_4_) as an oxidant in concentrated sulfuric acid medium, while Staudenmaier’s method uses potassium chlorate (KClO_3_) in a mixture of concentrated nitric and sulfuric acids. Brodie’s method uses KClO_3_ as an oxidant in fuming nitric acid medium [99]. GO has a 2D structure (Figure 6) that is derived from graphene. In addition to the intact sp^2^-hybridized carbons, due to the introduction of oxygen atoms, GO contains sp^3^ carbons as well. The presence of oxygen-containing functional groups such as -OH, -COOH, and epoxide groups on the surface makes GO hydrophilic and provides its optical and electronic properties. The COOH group on the surface allows covalent acylation, esterification, and amidation reactions for functionalization on both sides of the graphene sheets. Effective cross-linking of GO sheets can also be achieved by the nucleophilic opening of the epoxide ring. Other methods for functionalization of graphene include atom transfer radical polymerization, cycloaddition reaction, click reaction, and carbene insertion reactions [100]. GO is characterized by ease of surface modification, accessibility of both sides of the structured sheet for drug molecules, and the ability to make both covalent and non-covalent π–π stacking, H-bonding interaction, and electrostatic interaction with cargo molecules.

GO has an ultra-high surface area and is reported to load cargos such as drugs, cell-targeting ligands, nucleic acid, and proteins efficiently. These covalent and non-covalent modifications of GO are responsive to pH, temperature, UV and visible light, and electrical fields, providing GO with “smart” tumor-responsive properties. During the past few years, several groups reported the application of GO in drug delivery and cancer therapy [101,102]. GO itself is shown to exert apoptosis in human liver cancer cells (HepG2 cells) and could be explored for the treatment of human hepatocellular carcinoma (HCC) [103]. In another study, resveratrol rGO was reported to cause dose-dependent cytotoxicity in A2780 ovarian cancer cells [104]. Multifunctional GO nanocomposites have also been extensively used for both in vitro and in vivo photothermal and photodynamic applications for cancer treatment [105]. Since the GO structure consists of long-chain aromatic pi-electron clouds, the delocalization of the aromatic pi-electron cloud in GO enables absorption in the NIR (650–2500 nm) region [106]. The absorbed radiation is transformed into heat, leading to photoablation of the cancer cells and subsequent cell death. GO is also fabricated as carriers for photosensitizers and is shown to increase the selectivity and efficacy of PDT.

The unique planar π–π domains of graphene, the presence of COOH groups on the edges, and defects in the sheet allow electrostatic and charge exchange interactions with biomolecules and enable fabrication of GO as cellular imaging probes and biosensing agents [107]. One of the increasingly used applications of graphene and graphene-based materials is the development of electrochemical, electronic, and optical biosensors [108,109]. The fluorescence quenching ability of GO makes them valuable in the design of field-effect transistor (FET) biosensors [110]. For example, chemically modified graphene nanostructures are fabricated as biological devices for label-free detection of biomolecules, including nucleic acid, protein, cells, cell signaling molecules, and live-bacterium [111,112,113]. GO can be used for the immobilization of proteins without the need for additional surface modifications and cross-linking agents [114]. Further, both DNA and protein can be functionalized on graphene in tandem. For example, horseradish peroxidase was immobilized on a single-stranded DNA/graphene nanocomposites for direct electrochemistry and fabrication of electrochemical biosensors [109].

### 4.1. Polymer-Functionalized Graphene Oxide Nanoparticles

GO is water-soluble, but it often aggregates in the physiological buffer in the presence of salts and serum components. GO can induce dose-dependent cytotoxicity by inducing reactive oxygen species (ROS) production and oxidative stress [103,115]. To overcome these concerns, GO is often functionalized with polymers, including chitosan (CS) [116], polyethylene glycol (PEG) [44], polyethylenimine (PEI) [117], polyacrylic acid (PAA) [118], and polyvinyl alcohol (PVA) [119], among others. These surface-modified nanoparticles exhibit higher stability and biocompatibility, and improve the solubility of GO in water and physiological medium. The coating of the polymeric matrix prevents damage to cell membranes that usually occurs from the sharp edges of GO upon entry of nanoparticles into the cells. After the initial report on the PEGylation of nanographene oxide (NGO), several studies on the functionalization of GO as a nanocarrier for drug or gene delivery have recently been reported [120]. We will next provide a comprehensive account of functionalized GO and GO–metal nanoparticle nanocomposites for targeted drug delivery and cancer therapy.

#### 4.1.1. Chitosan-Functionalized GO Nanoparticles

CS is an amino polysaccharide, a hydrophilic, biocompatible, non-toxic, and biodegradable polymer of glucosamine and acetylglucosamine [121,122,123,124]. CS has been reported to have broad applications, including in biomedicine, agriculture, water treatment, food packaging, dentistry, ophthalmology, catalysis, textiles, paper, and biotechnology [125]. There are several reports on the surface modification of GO-based nanomaterials with CS for drug delivery applications [126]. CS is a mucoadhesive, non-toxic, biocompatible, and biodegradable natural polymer that, in combination with GO nanoparticles, enhances the strength and stability of GO–CS composite. The synthesis of GO–CS involves amide coupling between the COOH group on the GO and the amino group of CS. The addition of CS improves the solubility of GO sheets in acidic media. Further, GO–CS results in changes in particle size and zeta potential as a function of pH. The GO–CS displays greater dispersibility in phosphate-buffered saline and cell culture medium and has greater loading efficiency than PEGylated nanoscale GO.

Rana et al. reported CS-functionalized GO in which the amino group of the glucosamine sugar of CS was covalently bonded to the COOH group of GO via standard amide bond reaction [127]. The coupling reaction and exfoliation of functionalized GO–CS nanoparticles were confirmed by Fourier transform-infrared spectroscopy (FTIR), thermo gravimetric analysis (TGA), and X-ray photoelectron spectroscopy (XPS) analysis. Further, the authors reported a change in the physical appearance of GO after functionalization with CS. GO dispersion before functionalization was light brown; however, GO–CS dispersion was black. This color change may suggest the reduction of GO after covalent functionalization with CS. The CS-functionalized GO (FGOC) nanoparticles were stable for up to a few months. Negative charges of the terminal COOH group of GO and positive charge of protonated NH_2_ group of CS created electrostatic attraction, which is responsible for the stability of CS-functionalized GO dispersion. The authors pioneered CS-functionalized GO for controlled in vitro drug release of ibuprofen (IBU) and 5-fluorouracil (5-FU), which characteristically contain only one aromatic ring in their structures. IBU and 5-FU were loaded onto the GO–CS system via physisorption. IBU can produce hydrophobic and π–π stacking interactions, and an aromatic ring containing 5-FU is involved in π–π interactions with the aromatic basal planes on FGOCs. The authors also evaluated the cytotoxicity of 5-FU-loaded CS-functionalized graphene oxides (FGOCs) in CEM human lymphoblastic leukemia and Michigan Cancer Foundation-7 (MCF-7) human breast cancer cell lines.

Deb et al. reported the synthesis of folic acid (FA)-conjugated GO–CS nanocomposite [128]. The GO and CS solutions were mixed, ultrasonicated, and stirred overnight to make GO–CS composite. Next, FA was conjugated to the GO–CS via N-hydroxysuccinimide (NHS) and 1-Ethyl-3-(3-dimethylaminopropyl)carbodiimide (EDC) coupling, followed by sonication. The addition of CS and FA to GO was confirmed by FTIR and XRD studies. UV–VIS studies showed a peak of GO only at 230 nm, GO–CS at 224 nm and 300 nm, and GO–CS–FA at 248 nm and 371 nm. Scanning electron microscope (SEM) images showed a highly wrinkled surface of GO sheets, which were smoothened after functionalization. Transmission electron microscopy (TEM) images suggested the size of GO to be a few hundred nanometers, whereas functionalized GO–CS–FA size was in the micrometer range. The GO–CS composite attached to FA was used for in vitro targeted co-delivery of antitumor drugs, camptothecin (CPT), and bis(31-indolyl)methane (or 3, 31-diindolylmethane (DIM)). The resulting nano-biocomposites demonstrated a synergistic effect against the MCF-7 cell line [128].

In another study, Abbasian et al. reported the functionalization of GO nanosheets with a CS-graft—poly (methacrylic acid) graft (CS-g-PMAA) copolymer [129]. The synthesis of the CS-grafted-poly (methacrylic acid)/GO nanocomposite system was characterized through an FTIR, TGA, and NMR study. The authors first synthesized a CS chain transfer agent macroinitiator using 4-cyano,4-[(phenylcarbothioyl) sulfanyl] pentanoic acid as a chain transfer agent. The macroinitiator was then copolymerized with methacrylic acid using a reversible addition–fragmentation chain transfer polymerization method to produce CS-graft-poly(methacrylic acid) (CS-g-PMAA) graft copolymer. Next, GO sheets were incorporated into the CS-g-PMAA polymer to give the CS-g-PMAA/GO nanocomposite, followed by the loading of the anticancer drug doxorubicin (DOX). UV–VIS spectroscopic study of GO and CS-g-PMAA/GO nanocomposite revealed a characteristic peak of GO at 237 nm and a hump at 303 nm, which corresponds to π–π* transition and n–π* transition. After functionalization with the CS-g-PMAA copolymer, both π–π* transition and n–π* peaks were shifted to blue wavelength. SEM morphology studies revealed that after functionalization with CS-grafted poly(methacrylic acid), the smooth surface of GO transformed to wrinkled and crumpled nanosheets. The resulting nanocomposites were loaded with the DOX, and the DOX-loaded nanosystem was evaluated for its biocompatibility, pH-dependent release behavior, and cytotoxic effect against MCF-7 cell lines [129].

Lei et al. synthesized a drug delivery system comprising GO modified with CS and sodium alginate (SA) through electrostatic self-assembly. The nanocomposite was loaded with DOX through π-π stacking and electrostatic interactions. The synthesis of nanocomposite involved layer-by-layer self-assembly, in which the positively charged CS produced electrostatic interactions with the negatively charged GO and sodium alginate. Attachment of both CS and SA was confirmed with FTIR. Atomic force microscopy (AFM) analysis showed that the thickness of GO, GO–CS, and GO–CS–SA composites were 2 nm, 6 nm, and 60 nm, respectively. The GO–CS/SA nanocomposites were loaded with DOX. Further, a fluorescent label, fluorescein isothiocyanate (FITC), was attached to monitor the cellular uptake of DOX. GO–CS–SA–DOX displayed remarkable cytotoxicity attributed to the DOX’s faster release in response to the intracellular acidic pH [130].

Liu et al. designed another biocompatible nanocarrier comprising GO covalently linked to CS that was further decorated with γ-polyglutamic acid (γ-PGA). The GO–CO–γ-PGA composites were synthesized via an amide coupling reaction following the activation of the carboxyl groups of GO and γ-PGA with EDC and NHS and the reaction of the activated esters with the amino groups of chitosan oligosaccharide (CO). The functionalization was confirmed via FTIR and XPS studies. GO displayed UV–VIS peak at 230 nm; GO–CO had absorption peaks at 211, 230, and 301 nm; while GO-CO-γ-PGA showed peaks at 203, 230, and 301 nm. TEM and AFM studies revealed that GO–CO–γ-PGA had a size of 200–300 nm and thickness of around 7–8 nm. The authors evaluated the GO–CO–γ-PGA composite for pH-dependent controlled release of DOX into HeLa cells. While the GO–CS–γ-PGA nano-drug composite exhibited noteworthy cytotoxicity to HeLa cells, no cytotoxicity against normal cell lines was observed [121].

Zhao et al. reported another GO–CS-based drug vehicle system for the delivery of DOX to HepG2 cells [131]. The drug carrier consists of CS and dimethyl maleic anhydride (DMMA)-functionalized GO nanoparticles. The COOH group of GO was reacted with the NH_2_ group on CS, and then the GO was coated with an outer layer of DMMA. Total internal reflection fluorescence (TIRF) microscpy and NMR confirmed the addition of CS–DMMA to GO. Zeta potential values of GO nanoparticles changed from −42.3 mV to 36.4 mV after surface functionalization with the positively charged CS. The zeta potential further changed to −26.5 mV after functionalization with DMMA. TEM images revealed that the size of GO increased from 84 nm to 114 nm after surface functionalization. Further, DOX was loaded via π–π stacking interactions between the conjugated structures of GO and the aromatic rings of DOX. The CS-based system served as a smart protecting screen that prevents the premature release of DOX in normal extracellular conditions. However, at low pH, site-specific drug release was achieved after the removal of the CS–DMMA layer. The GO–CS–CS–DMMA nanocarrier also displayed an enhanced therapeutic effect against the human liver HepG2 cancer cell line [131].

SreeHarsha et al. designed a GO–CS-based drug vehicle for the delivery of DOX to PC-3 prostate cancer cell line [123]. GO was first prepared by Hummer’s method, followed by its reduction to rGO. GO showed UV–VIS peak at 230 nm, whereas rGO showed UV peak at 270 nm. The rGO has a size of 340.55 ± 21.78 nm and a zeta potential of −35.1 ± 3.4 mV. The DOX was loaded to rGO hybrid by incubation for 24 h to afford the DOX-loaded rGO hybrid nanoparticle (rGOD–hNP). The rGOD–hNP composite was finally coated with 0.1% *w*/*v* CS and stabilized with thiamine pyrophosphate (TPP) (0.125% *w*/*v*) to give TPP-stabilized rGOD–hNPs. The rGO had a size of 340.5 nm, which, after functionalization with CS and TPP, increased to 520.5 nm. This drug carrier demonstrated high biocompatibility and efficient drug loading (65%) and release (50% in 48 h) and offers a promising approach to deliver DOX for PTT of prostate cancer [123].

Xie et al. reported the pH-dependent controlled release of DOX in the MCF-7 cell line using CS and dextran (Dex)-functionalized GO nanoparticles [124]. Functionalization of GO with CS and Dex was achieved through a non-covalent self-assembly technique using electrostatic and hydrophobic interactions. The incorporation of CS and Dex was achieved through electrostatic interactions in two steps. First, positively charged CS was reacted with the negatively charged GO to obtain the CS-modified GO. In the next step, the negatively charged CS was successfully deposited. The addition of CS and Dex improved GO’s solubility in the physiological buffer and reduced non-specific protein adsorption of GO sheets. The GO-CS/Dex nanocomposites were evaluated for the delivery of DOX. The CS and Dex-modified GO demonstrated efficient cellular uptake and localization in the cytoplasm of the cells and exhibited dose-dependent cytotoxicity to MCF-7 and HepG2 cell lines [124]. We summarize in Table 1 the different GO–CS composites developed as nanocarriers for drug delivery in cancer therapy.

#### 4.1.2. PEG-Functionalized GO Nanoparticles

Coating a therapeutic with a “stealth” polymer such as PEG protects it from degradation and increases the circulating time of NPs in the bloodstream. PEG is a polymer of repeating ethylene ether units that is widely used for several pharmaceutical and biomedical applications. Due to its safety, it is classified as “Generally Regarded as Safe” (GRAS) by the FDA. The first FDA-approved PEGylated protein product, Adagen, became available in 1990 as an enzyme replacement therapy for the treatment of severe combined immunodeficiency disease. Subsequently, PEG was extensively explored for more effective intravenous delivery of nanoparticle-mediated therapeutics [59]. PEG could be linked to nanoparticles by either covalent or non-covalent methods. Coating nanoparticles with PEG masks the charges on nanoparticles and reduces their aggregation, opsonization, and clearance by macrophages. PEGylation is also reported to reduce immunogenicity and hemotoxic effects associated with nanoparticles. The ethylene glycol units of PEG are hydrophilic and increase the solubility of associated nanoparticles in buffers and serum [132,133,134,135,136,137].

In 2008, Sun et al. reported the synthesis and biomedical applications of PEGylated single layer NGO with a width of a few nanometers [120]. The authors prepared NGO with lateral dimensions <10 nm through sonication. PEGylated NGO was synthesized by grafting six-armed PEG-NH_2_ to activated GO–COOH, which was made by converting the OH groups to COOH moieties and activating the epoxide and ester groups of GO with chloroacetic acid under basic pH. After PEGylation, AFM analysis showed the NGO–PEG sheets were mostly <20 nm in size. GO sheets showed absorbance peak at 230 nm; however, absorbance spectra of PEG-modified NGO shifted towards the NIR region. The absorbance of PEG-modified GO was 480%, 780%, and 470% higher than GO absorbance at wavelengths of 500 nm, 808 nm, and 1200 nm. GO sheets gave fluorescence maxima at 570 nm, which became blue-shifted to 520 nm after PEG functionalization. These PEG-functionalized NGO were soluble in physiological buffer and serum without any aggregation. Further, their intrinsic visible and NIR fluorescence property remained intact after PEG functionalization. The photoluminescence of NGO was used for live cancerous cell imaging of CEM.NK T cells and Raji B cells in the NIR region. For selective targeting of cancer cells, the B cell-specific antibody Rituxan was attached to PEGylated NGO through a sulfosuccinimidyl 4-N-maleimidomethyl cyclohexane-1-carboxylate (sulfo-SMCC) linker. Further, this PEG–NGO–Rituxan combination was successfully used for precise and selective in vitro drug delivery of DOX to CEM.NK T cells and Raji cell lines [120].

Ma et al. fabricated a PEG functionalized super-paramagnetic GO-iron oxide nanoparticle (GO–IONP) nanocomposites for cancer therapy [44]. GO was synthesized from graphite using the modified Hummer’s method. Next, the GO–IONP composite was synthesized by mixing GO, FeCl_3_, sodium acrylate, and sodium acetate in ethylene glycol and diethylene glycol as a solvent medium. PEG was added to GO–IONP nanocomposite by activation with EDC to make PEG–GO–IONP composite. TEM images suggest the size of PEGylated GO–IONP nanocomposite was around 10 nm, while the as-made GO size was 50–300 nm. However, AFM images suggested a significant increase in the thickness of GO–IONP–PEG sheets compared to GO after functionalization. To evaluate the magnetically targeted drug delivery of nanocomposites, DOX was loaded onto the GO–IONP–PEG through π–π stacking interactions between and the GO and DOX. The GO–IONP–PEG–DOX complex was used for in vitro delivery of DOX to murine breast cancer 4T1 cells. The carrier’s drug loading efficiency enhanced from 44% to 220%, with increasing concentration of DOX loading. A pH-dependent drug release was observed in this system. At pH 7.4, 20% DOX released from the nanocarrier and, at pH 5, 50% DOX released within a time scale of 360 min. A 3-(4,5-dimethylthiazol-2-yl)-2,5-diphenyl tetrazolium bromide (MTT) cell viability study suggested that cytotoxicity of GO–IONP–PEG–DOX was similar to free DOX. Moreover, GO–IONP–PEG was also utilized for magnetically targeted PTT using the nanocarrier’s strong absorbance in the NIR region. Finally, this innovative nanocarrier was successfully applied for in vivo magnetic resonance imaging of breast cancer tumor-bearing mice, where the GO–IONP–PEG nanocomposites acted as a contrast agent [44].

Recently, Kazempour et al. described surface modification of GO with PEG-4000 for delivery of DOX to A549 adenocarcinoma human alveolar-basal epithelial cells [138]. First, GO was synthesized using the Hummer’s method and modified Offeman method. Next, the GO was converted to GO chloride by thionyl chloride, and then the GO–Cl was reacted with PEG 4000 in dimethylformamide. SEM images suggest a wrinkled surface of GO sheets, but the surface of GO got smoothened after functionalization with GO-4000. The GO–PEG-4000 nanocarrier was loaded with DOX with a drug loading efficiency of 87%. The drug release behavior was pH-dependent and occurred in two steps. In the first step, a rapid discharge occurred, in which 2.5% and 3.5% of drugs were released at pH 7.4 and 5.8, respectively, on a 1-h time scale. The authors claimed that in this first step, drug molecules attached to GO–PEG surface via hydrogen bonding were released. In the second step, the continuous discharge was observed for 3 h at both pH values. The authors explained that in the second step, drug molecules attached via π–π stacking were released. Further, MTT assays suggested lower cytotoxicity of GO–PEG compared to GO. Thus, this GO–PEG-4000 nanocarrier prevents GO from aggregating in the physiological medium and significantly reduces its cytotoxicity, which is evident from the half maximal inhibitory concentration (IC_50_) values [138].

Pei et al. demonstrated the co-delivery of two drugs, cisplatin (Pt), combined with DOX using four-armed PEG-functionalized NGO for enhanced synergistic therapeutic effect [139]. The synthesis first involves the preparation of PEGylated GO. Next, a polymeric Pt prodrug containing the carboxylic group was covalently attached to the amino groups of PEGylated GO nanosheets (pGO) via EDC- and NHS-mediated amide coupling reaction. Subsequently, DOX was loaded onto pGO–Pt through non-covalent π–π interactions to afford the dual-drug delivery system (pGO–Pt/DOX). The mean size of PEGylated GO was found to be 146.1 nm. Zeta potential of GO changed from −36.8 mV to −16.8 mV, indicating the neutralization of some of the –COOH groups of GO and coupling of PEG. This dual drug delivery to tumor cells induced an apoptosis and necrosis rate of 18.6%, which was almost two times higher than that of the individual pGO–Pt or pGO–DOX groups. The cytotoxicity of PEGylated GO/Pt/DOX to healthy organs diminished to some extent more than cytotoxicity caused by Pt or DOX when loaded individually. Interestingly, Pt was attached to PEGylated GO through covalent binding, and DOX was loaded onto PEG–GO/Pt by π–π stacking. The weight ratio of DOX/Pt/PEGylated GO was optimized to 0.376:0.376:1 for efficient drug loading. The drug release behavior was studied at two different pH values. At pH 7.4, Pt and DOX’s release was 28.4% and 29.1%, respectively, over 72 h. At pH 5.3, the amount of Pt and DOX released was 30% and 41%, respectively, in the first 4 h, which increased to 65.7% and 64.6%, respectively, in 72 h [139].

**Figure 7:**
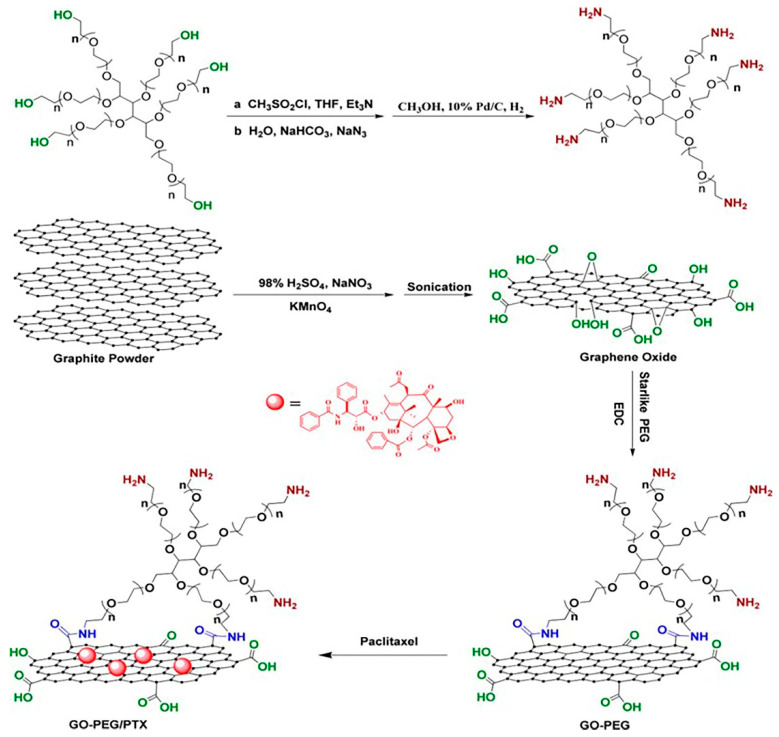
Preparation of graphene oxide (GO)–polyethylene glycol (PEG)/paclitaxel (PTX) nanoscale drug delivery system [140]. (Reprinted with permission from Zhiyuan Xu et al. ‘Covalent Functionalization of Graphene Oxide with Biocompatible Poly(ethylene glycol) for Delivery of Paclitaxel’. *ACS Appl. Mater. Interfaces* **2014**, *6*, 17268−17276. Copyright (2020) American Chemical Society).

Xu et al. developed a PEG-functionalized GO (Figure 7) for delivery of anticancer drug paclitaxel (PTX) [140]. First, GO was synthesized by modified Hummer’s method followed by filtration and subsequently dialysis and water-bath ultrasonication to obtain nano-sized GO that would not aggregate for several months. The functionalization of GO was carried out with a six-armed PEG–NH_2_ through a covalent amide-coupling reaction. The reaction was confirmed by the absence of a sharp peak at 1732 cm^−1^ and the appearance of an amide (-NH–CO-) peak at 1652 cm^−1^ in FTIR spectrum. AFM images suggest that the thickness of PEG–NH_2_-grafted GO increased from 0.8–1.4 to 1.6–3.5 nm. AFM images also revealed that the smooth surface of GO became disturbed after functionalization. UV–VIS spectra of PEG–NH_2_-modified GO covered the range 200–700 nm without a distinct absorption peak. The GO–PEG nanocarrier was found to be stable in the biological medium. Covalent attachment of a fluorescent probe to the GO–PEG complex enabled intracellular imaging and showed the GO–PEG carrier’s uptake into cells. The GO–PEG by itself was nontoxic to human lung cancer A549 and human breast cancer MCF-7 cells. GO–PEG was conjugated to PTX via π–π stacking and hydrophobic interactions. The design of the GO–PEG–NH_2_ nanocarrier and loading of drug PTX is explained in Figure 7. The drug loading capacity for this nanocarrier was 11.2 wt%. The GO–PEG–PTX nano complex exhibited significant cytotoxicity to A549 and MCF-7 cells compared to the free drug, PTX. However, the authors did not investigate the drug release behavior in this work [140].

Tian et al. reported a PEGylated FA and peptide-modified GO as a nanocarrier for targeted delivery of CPT [141]. The synthesis involved conjugation of a fluorescein-labeled caspase-3-specific substrate peptide (CALNNDEVDK-FAM, Pep-FAM) on to the surface of GO. Subsequently, the PEGylated FA was conjugated to the peptide-modified GO. AFM studies confirmed the dimension of GO sheets to be around 100 nm. After surface functionalization, the topological height of GO sheets increased to 7.5 nm. Absorption spectra of FA–PEG–GO showed a peak at 280 nm; FA–pep–PEG–GO showed absorption peak maxima at 230 nm, 445 nm, and 480 nm. Zeta potential of FA/pep/GO was −20.7 mV. The authors then loaded CPT, an aromatic and hydrophobic anticancer drug, on the nanovehicle. The drug loading capacity of 1.7 mg/mg and a loading efficiency of 90% was achieved. Drug release was pH-dependent. At physiological pH 7.4, 21.5% CPT was released from pep–FA–PEG–GO nanocarrier in 48 h, whereas at acidic pH 5.0, 71% of the drug was released from nanocarrier at the same time. IC_50_ values suggested that cytotoxicity of free CPT and pep–FA–PEG–GO–CPT was comparable.

Miao et al. reported PEGylated GO as a nanocarrier for co-delivery of photosensitizer chlorin e6 (Ce6) and DOX to explore their synergistic anticancer effect [142]. First GO was prepared using the Hummer’s method with slight modification, followed by exfoliation by sonication to obtain the GO nanosheets. PEGylated GO was synthesized using standard covalent amide coupling reaction between PEG–NH_2_ and GO in the presence of EDC and NHS, and subsequently, Ce6 and DOX were loaded onto pGO nanosheets. AFM studies revealed that the pGO nanosheets were 1 nm thick, while the size of the Ce6- and Dox-loaded pGO (Ce6/Dox/pGO) was 148 nm. The polydispersity index of PEGylated GO was determined to be 0.21. Dynamic light scattering (DLS) zeta potential measurements suggested surface charge of PEGylated GO nanosheets was −41.5 mV. Loading efficiency using this nanocarrier was calculated to be 51.9% for DOX and 61.7% for Ce6. Zeta potential measurements suggested that surface charge of a drug-loaded nanocarrier was comparable to that of the nanocarrier only without any drug. Difference combination index (Cl) values calculated by the Chou–Talay method suggested that Dox:Ce6::1:8 did not give rise to any synergistic anticancer effect, however, Dox:Ce6::1:2 and Dox:Ce6::1:4 resulted in a strong synergistic anticancer effect. We summarized in Table 2 the different GO–PEG composites developed as nanocarriers for drug delivery in cancer therapy [142].

#### 4.1.3. Hyaluronic Acid-Functionalized GO Nanoparticles

Hyaluronic acid (HA) is a naturally occurring, biocompatible, non-immunogenic, and biodegradable polysaccharide, consisting of alternating units of d-glucuronic acid and *N*-acetyl-d-glucosamine [143]. HA is capable of precisely recognizing the transmembrane glycoprotein CD44 receptors that are enormously expressed on tumor cell surfaces [144,145,146,147,148]. HA has good stability; it is cost-effective and has good aqueous solubility. HA has OH and COOH groups that can be chemically modifiable and covalently linked to anticancer drugs through amide and ester linkages. Over the past decade, several research groups have used HA in drug delivery applications [149,150,151].

**Figure 8:**
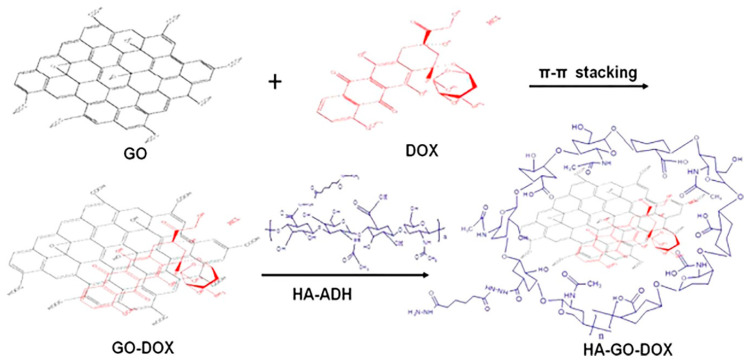
Schematic illustration of the preparation of a hyaluronic acid (HA)−graphene oxide (GO)−doxorubicin (DOX) nanohybrid [151]. (Reprinted with permission from Song et al. ‘Hyaluronic Acid-Decorated Graphene Oxide Nanohybrids as Nanocarriers for Targeted and pH-Responsive Anticancer Drug Delivery’. *ACS Appl. Mater. Interfaces* **2014**, *6*, 11882–11890. Copyright (2020) American Chemical Society).

Song et al. reported HA-modified GO as a nanocarrier (Figure 8) for the delivery of DOX in vitro and H22 hepatic cancer cell-bearing mice [151]. The synthesis involved the loading of DOX onto GO nanoparticles through π–π stacking and H-bonding interactions. HA was modified with adipic acid dihydrazide (ADH), and HA–ADH obtained was then coated onto GO–DOX via H-bonding between the amine group of HA–ADH and epoxy groups in GO to afford nanohybrids. GO sheets gave a characteristic absorption peak at 230 nm, while the coating of HA–ADH resulted in a slight decrease in absorbance. AFM images suggested that the size of GO sheets was 10–200 nm and thickness was less than 2 nm, while surface modification increased the thickness of GO. The drug loading ratio using this nanocarrier was satisfactory (42.9%). Drug release at physiological pH 7.4 was less than 20% at 24 h, whereas at acidic pH 5.3, the drug release rate was 40% at 24 h. Cellular uptake and release of drugs using this system were time- and dose-dependent. HA–GO–Dox showed more cytotoxicity towards HepG2 cells compared to free Dox and GO–Dox. The authors showed that the HA–GO–DOX inhibited tumor growth in mice in a dose-dependent manner, with a 6 mg/kg dose found to be most effective [151].

Jung et al. synthesized an HA-modified nanographene composite for the targeted delivery of epirubicin [152]. The authors synthesized HA–NGO by conjugating 38% hexamethylenediamine-modified HA with the carboxylated NGO. The TEM image of HA–NGO showed a spherical morphology with an average size of 250 nm. The authors tested the HA–NGO nanoconjugate against B16F1 cancer cell lines that overexpress HA receptors, CD44, and lymphatic vessel endothelial hyaluronan receptor 1 (LYVE-1). The drug epirubicin was loaded on GO–HA conjugate through π–π stacking interactions. The cellular uptake of the epirubicin/NGO–HA complex was mediated via the HA receptor-facilitated endocytosis. With the initial epirubicin concentration of 0.27 mg/mL, the drug loading efficiency on NGO–HA was 2%, 9%, and 25% at pH 4, 7, and 9, respectively. The in vitro drug release from the epirubicin/NGO–HA complex was also pH-dependent. At pH 5, 50% drug release occurred in the first 24 h, followed by 70% drug release in 72 h. In contrast, at pH 7, only 18% of drug release occurred in 72 h. The pronounced release of an anticancer drug under acidic conditions is advantageous for specific targeting and delivery of the drug into the tumor cell. Thus, this NGO–HA–epirubicin combination can be effectively applied for the treatment of tumors overexpressing HA receptors [152].

Liu et al. synthesized an innovative redox-responsive nanocarrier system based on GO–HA [153]. The synthesis involved the reaction of activated HA with cystamine dihydrochloride containing disulfide bonds (SS) to obtain the HA–SS–NH_2_ complex. Next, the NHS- and EDC-activated NGO was reacted with HA–SS–NH_2_ to achieve the NGO–SS–HA composite, where the HA with NGO was conjugated via a linker comprising disulfide (-SS-) bonds. AFM and TEM studies revealed that the thickness of GO increased from 1 nm to 2.5 nm after functionalization. However, the size of the modified GO decreased from 250 nm to 125 nm after functionalization. Cellular uptake of NGO–SS–HA was mediated by CD44 receptors on the human lung carcinoma A549 cells. Further, this nanocarrier was used for the delivery of the anticancer drug gefitinib (Gef). The NGO–SS–HA showed specific binding to cancer cells and exhibited redox-responsive cargo release in tumor cells overexpressing GSH. The authors investigated the therapeutic efficacy of NGO–SS–HA–Gef in vitro on A549 cell lines and in vivo in lung cancer cell-bearing mice.

Wu et al. synthesized modified NGO and introduced amino groups by activating GO with EDC and NHS and reacting the activated GO with adipic acid dihydrazide (ADH) [154]. FTIR of GO–ADH showed the disappearance of the peak at 1725 cm^−1^, with a new band appearing at 1630 cm^−1^, indicating the formation of the amide bond in GO–ADH. Next, HA was attached to GO–ADH via amide coupling. This GO–adipic acid–HA conjugate was successfully applied for both in vitro and in vivo delivery of DOX. TEM and AFM studies revealed that GO–HA had a thickness of 2–5 nm, where GO had a thickness of 0.8–1.3 nm, and the size of GO–HA decreased from 60–500 nm to 40–350 nm after functionalization. The GO–adipic acid–HA-conjugated system was adequately taken into tumor cells by receptor-mediated endocytosis. The GO–HA by itself displayed insignificant hemolytic activity. MTT assays suggested reasonably low cytotoxicity of this system towards both human cervical cancer HeLa cells and the L929 murine fibroblast cell line. Further, this system also showed negligible acute toxicity towards mice. The GO–adipic acid–HA nanocarrier system was loaded with DOX and achieved a maximum loading ratio of 81.5%. This nano-drug carrier can selectively target tumors as the drug is preferentially released under acidic pH. For example, the drug was released insufficiently at pH 6.3 and 7.4 in 65 h. However, at pH 5.2, the drug’s release improved significantly to 26% in 65 h. The GO–adipic acid–HA–DOX therapeutic system was further extended in vivo using the cervical HeLa tumor model on nude mice. GO–adipic acid–HA–DOX therapy suppressed the tumor growth by 40% by day 16, without any significant side effects. Thus, this GO–adipic acid–HA–DOX was developed as a targeted drug delivery system that inhibits tumor growth, both in vitro and in vivo.

Li et al. developed HA–GO composites using ADH as a linker and Ce6 as a photosensitizer (PS) for targeted and photoactive switching-enabled PDT to HeLa cells and mouse embryonic fibroblast NIH3T3 cells [155]. The authors first prepared fractionated GO sheets of size <100 nm by repetitive sonication for a longer duration. ADH was attached to HA through EDC-mediated covalent amide coupling. Next, GO–ADH–HA conjugates were synthesized by reacting the amino groups of the HA–ADH with the carboxylic acid groups on pristine GO sheets through an amide coupling reaction, and the conjugation was confirmed by FTIR, UV, and TGA analysis. DLS studies revealed that the size of GO increased from 59.3 to 78.1 nm after surface modification with HA. AFM studies suggested that the thickness of GO–HA increased from 1 to 2.8 nm. UV spectra of GO–HA slightly red-shifted compared to GO only. Finally, Ce6 was loaded onto GO–ADH–HA conjugates via π–π stacking and hydrophobic interactions. This GO–ADH–HA–Ce6 nanohybrid was internalized by cells more effectively than compared to free Ce6. The presence of HA in the composite system enabled targeted delivery to cancer cells. Interestingly, the photoactive nature of Ce6 adsorbed onto GO–ADH–HA diminished in water or physiological buffer. However, upon cellular uptake, the photoactivity of Ce6 recovered rapidly after release from the GO–ADH–HA nanocarrier. The authors found that the GO–ADH–HA/Ce6 composite system’s photodynamic efficiency was almost 10 times better than the photodynamic efficiency of Ce6 only.

Guo et al. developed a dual-receptor-targeting drug delivery system, in which GO was functionalized with HA via ADH linker, and further with Arg–Gly–Asp peptide (RGD) for targeted drug delivery with more selectivity and specificity [156]. The synthesis first involves the modification of HA with ADH to introduce an amino group on the HA’s surface. Next, HA–ADH reacted with the COOH on the edges of GO through the EDC- and NHS-mediated amidation reaction. Subsequently, the 4-Aminophenylmercuric acetate (APMA) linker was introduced on the GO to obtain methacrylated GO–HA, which was finally reacted with cysteine-containing CGRGDSY peptide to obtain the GO–HA–RGD conjugate. TEM images suggested that the size of GO–HA–RGD was in the range of 70–490 nm. AFM study revealed that the thickness of GO sheets increased from 1.2 to 5.7 nm and 13 nm after conjugation with HA and RGD, respectively, wherein the GO surface was flat, but the GO–HA–RGD surface was coarse. This GO–HA–RGD system was successfully applied for in vitro delivery of DOX to human Caucasian ovary adenocarcinoma (SKOV-3) cell lines. The drug loading efficiency of 72.9% was achieved using a GO/DOX weight ratio of 1:1. At the stimulated tumor microenvironment (pH 5.5), the drug release rate was 30.2%. However, at the physiological condition (pH 7.4), the drug release rate was only 7.6%. The GO–ADH–HA–RGD nanocarrier suggested no apparent cytotoxicity to SKOV-3 and human ovarian surface epithelial cells (HOSEpiC) cell lines in an MTT assay. Interestingly, cytotoxicity of GO–HA–RGD–DOX (two-receptor) was higher than GO–HA–DOX (one-receptor) or GO–DOX (no receptor) for SKOV-3 cell lines. Cellular uptake study revealed higher cellular uptake of GO–HA–RGD–DOX compared to GO–HA–DOX or GO–DOX systems. This specific dual targeting ability was achieved through the combined effect of the CD44–HA receptor, and integrin–RGD-facilitated endocytosis [156].

Sousa et al. investigated the potential of rGO conjugated with HA for PTT [157]. rGO was synthesized by the reduction of GO with L-ascorbic acid. rGO generally exhibits low aqueous solubility, low cytocompatibility, and is less selective towards cancer cells. To overcome these problems, rGO was surface-modified using HA-based amphiphilic polymer with a targeting capacity for the CD44 receptors. HA was grafted onto poly(maleic anhydride-alt-1-octadecene) (PMAO) to give HA-g-PMAO. The functionalization of rGO with HA-g-PMAO involves simple mixing, sonication, and dialysis steps. DLS studies revealed that surface functionalization of rGO with HA-g-PMAO did not affect its size distribution. TEM studies suggested the size of the composite to be 108 nm. Zeta potential of functionalized rGO was −28.6 mV, whereas the zeta potential of rGO was only −26.9 mV. NIR absorption for both rGO and HA–rGO was similar, which suggests that the functionalization of rGO did not affect its photothermal property. Surface modification of rGO significantly increased its solubility and cytocompatibility, as well as cellular uptake by cancer cells overexpressing CD44 receptors. Further, this HA-modified rGO induced cancer cell ablation in MCF-7 cells when irradiated with NIR light at 808 nm. Thus, HA–rGO is a promising candidate for CD44-targeted PTT as a cancer treatment [157].

Recently, Pramanik et al. illustrated the application of HA-modified GO for in vitro dual drug delivery for efficient cancer therapy [158]. First, amino groups were introduced on HA by the reaction of ethylenediamine with HA. Next, GO was activated with EDC and NHS and reacted with the amino groups of modified HA through an amide-coupling reaction. Conjugation of HA on GO was studied by UV–VIS, XRD, FTIR, and Raman spectroscopy. DLS studies revealed that the mean hydrodynamic diameters of GO and GO–HA were 184 nm and 166.8 nm, respectively. Zeta potential of GO and GO–HA were −18 mV and −21.5 mV, respectively. TEM and AFM studies revealed the size of GO and GO–HA to be in the range of 130–160 nm and thickness in the range of 1–2.5 nm. The HA-functionalized GO showed low cytotoxicity to two breast cancer cell lines, namely, BT-474 and MDA-MB-231. In the present work, two anticancer drugs, DOX and PTX, were simultaneously loaded onto HA–GO conjugate. Selective targeting was achieved with the GO–HA–DOX–PTX system against MDA-MB-231 cells overexpressing CD44 receptors. Furthermore, FeO nanoparticles were coupled with the GO–HA–Dox system for magnetothermal therapy. The synergistic effect of magnetic hyperthermia combined with DOX therapy through GO–HA–Dox/FeO demonstrated improved efficacy in killing tumor cells, compared to GO–HA–Dox or FeO-induced magnetotherapy only [158]. We have summarized in Table 3 the GO–HA composites developed as nanocarriers for drug delivery in cancer therapy.

#### 4.1.4. PVA-Functionalized GO Nanoparticles

PVA is a water-soluble polymer synthesized from hydrolysis and radical polymerization of vinyl acetate [159]. It has excellent optical properties, large dielectric strength, and charge storage ability. PVA has found wide applications in fibers, films, optics, hydrogels, and biomaterials. PVA is used for biomedical applications due to its biocompatibility, low toxicity, and low propensity for protein adhesion. Chemical transformation of PVA involves pendant hydroxyl groups’ reactions through esterification, acetalization, carbamation, and etherification reactions [159]. Other transformations include the introduction of phosphoryl and functionalization of PVA with azido and alkyne groups for click chemistry [160,161].

Sahoo et al. reported the synthesis of PVA-functionalized GO for improving the aqueous solubility and biocompatibility of GO [119]. First, GO was synthesized using the modified Hummer’s method. PVA-functionalized GO was synthesized through a carbodiimide-activated esterification reaction. FTIR, TEM, and AFM confirmed the functionalization of PVA on the GO. The TEM images showed the size of GO–PVA composite to be in the range of 100–200 nm. AFM studies revealed the thickness of GO sheets increased from 0.8–1 to 2–3 nm after functionalization with PVA; the GO surface looked smooth, whereas the GO–PVA surface was coarse. The authors used GO–PVA to deliver CPT and explored the cytotoxicity of GO–PVA–CPT against human breast and skin cancer cells. The drug-loading behavior showed that 0.12 g CPT was loaded per 1 g GO–PVA. The drug release profile in PBS showed about 20.0% of CPT released in 72 h, suggesting strong π–π stacking and hydrophobic interactions between the GO–PVA and CPT. Further, cytotoxicity studies demonstrated that the GO–PVA–CPT conjugate system is much more toxic towards cancer cells than free CPT only, whereas there was no apparent cytotoxicity for GO–PVA without the drug. GO–PVA–CPT caused more than 50% inhibition of tumor growth in a human breast cancer cell line (MDA-MB-231) and metastatic skin tumor cell line (A-5RT3). Thus, PVA-functionalized GO represents a potential nano-drug carrier for chemotherapeutic agents [119].

#### 4.1.5. PAA-Functionalized GO Nanoparticles

PAA is a synthetic polymer of acrylic acid monomers. In aqueous solution, PAA has an anionic nature because of its carboxylic groups. PAA is a biocompatible, non-toxic, pH-sensitive, and mucoadhesive polymer.

Lu et al. reported the synthesis of PAA-grafted GO and explored the potential of PAA–GO as a nanocarrier for specific targeted delivery of 1,3-bis(2-chloroethyl)-1-nitrosourea (BCNU) for brain tumor therapy [118]. PAA was linked to the GO surface through a free-radical polymerization reaction. The synthesis of PAA-modified NGO involves mixing GO with acrylic acid monomers and potassium persulfate and running a free radical polymerization reaction, followed by centrifugation and washing steps. AFM studies revealed that GO and PAA-modified GO had similar lateral widths and dimensions. However, the thickness of PAA-grafted GO increased from 0.9 to 1.9 nm, and no impurity was seen on the surface of PAA–GO. PAA–GO displayed significantly excellent dispersibility and did not agglomerate in water after several months. XPS study showed PAA and COOH of GO to have a 1:1 peak ratio, proving successful incorporation of PAA into the GO surface. PAA surface functionalization improved thermal stability, solubility, and cell penetration capability of GO. Next, BCNU was immobilized on the surface of PAA–GO through an amide-coupling reaction. The drug-loading capability of PAA–GO increased with an increasing BCNU concentration, with a plateau achieved with 0.4 mg of the drug. At the saturation point, the maximum drug loaded on the nanocarrier was 200 μg BCNU per gram PAA–GO. However, drug activity diminished with increasing BCNU use in conjugation, which can be attributed to steric hindrance or a probable change in structure. Optimal conditions for BCNU immobilization onto PAA–GO resulted in a drug loading capacity of 198 μg BCNU/mg GO–PAA and 70% activity. In vitro confocal microscopy and TEM images confirm sufficient cellular uptake of PAA–GO conjugate by mouse glioma 261 (Gl261) cells. Cell proliferation XTT assay suggested no apparent cytotoxicity of PAA–GO alone towards GL261 cells. However, GO–PAA–BCNU was toxic to GL261 cells in a dose-dependent manner, with an IC_50_ of 18.2 µg/mL [118].

#### 4.1.6. PEI-Functionalized GO Nanoparticles

Zhang et al. reported GO with PEI functionalization and investigated the potential of GO–PEI in sequential delivery of Bcl-2-targeted siRNA and DOX [117]. The synthesis of PEI-modified GO involved covalent amide bond formation between GO and PEI in the presence of activator EDC. Elemental analysis showed 42.9% PEI was present in the PEI–GO composite. AFM studies revealed that the lateral dimension of GO did not change after modification with PEI, but thickness increased from 1–2 nm to 3–4 nm after surface modification with PEI. Zeta potential of PEI–GO composite was 55.5 mV, which supports adsorption or uptake of negatively charged cells or biomolecules. The cellular uptake of free RNA is limited because of significant negative charges on its surface. The present study incorporated cationic polymers for the loading of siRNA via electrostatic adsorption. siRNA was loaded on the PEI–GO vector by simple mixing of siRNA and GO–PEI in aqueous solution, and the siRNA-loaded GO–PEI exhibited strong cellular uptake into HeLa cells, as suggested by the confocal microscopy. The water-soluble tetrazolium salt (WST) assay showed that the PEI–GO composite has no apparent cytotoxicity. The authors also loaded the PEI–GO nanocarriers with DOX. The fluorescence signal from DOX was used to monitor the release of DOX from the PEI–GO. After loading of DOX onto PEI–GO, the fluorescence of DOX was completely quenched, and the fluorescence reappeared after the release of DOX in the cytoplasm of HeLa cells at 2 h. In contrast, the fluorescence was spotted in the nucleus at 6 h. Thus, PEI–GO–DOX was found to be stable inside cells, and very little loss of drug occurred before cellular uptake with this conjugate system. Finally, the authors investigated the effect of co-delivery of the Bcl-2-targeted siRNA and DOX to HeLa cells with PEI–GO as a nanocarrier. No synergistic effect was observed when siRNA and DOX were co-delivered. However, sequential delivery of GO–PEI–siRNA followed by GO–PEI–DOX resulted in significantly increased killing efficiency of the PEI–GO/DOX composite.

#### 4.1.7. Polyvinylpyrrolidone-Functionalized GO Nanoparticles

Polyvinylpyrrolidone (PVP) is a water-soluble, non-ionic, non-toxic polymer surfactant. It is synthesized from monomer N-vinyl pyrrolidone polymerization and contains pyrrolidone moiety of the hydrophilic and alkyl groups as the hydrophobic components. PVP is widely used in the synthesis of nanoparticles and as a capping agent in nanomedicine. PVP acts as a surface stabilizer, growth modifier, nanoparticle dispersant, and reducing agent. PVP is a phase transfer agent soluble in both aqueous and organic solvents [162]. The coating of PVP is reported to improve the dispersibility and biocompatibility of GO in physiological buffer [163].

In 2013, Qin et al. reported combined chemotherapy and PTT using FA conjugated to PVP-modified NGO [164]. FA conjugated GO–PVP composite was synthesized by activating NGO with chloroacetic acid and adding PVP and finally mixing FA with GO–PVP in the presence of EDC and NHS. TEM images suggest the size of GO–PVP less than 100 nm. UV–VIS absorbance spectra of NGO showed absorbance maxima at 232 nm, which was red-shifted to 250 nm for PVP-modified NGO, suggesting that the π–π-conjugated structure of GO was restored even after surface modification with PVP. The FA–NGO–PVP conjugate was tested for PTT, which was performed using a 2 W/cm^2^ power density laser light at NIR wavelength 808 nm. FA–NGO–PVP conjugate exhibited impressive photothermal energy conversion efficiency, even at a low concentration of 2.5 µg/mL. Further, for chemotherapy, DOX was loaded onto FA–NGO–PVP with a high loading ratio of 107.5 wt%. Drug release in PBS showed that at pH 7.4, only 13% DOX was released after 70 h of dialysis, whereas at pH 5.5, 60% of DOX was released after 70 h. Further, the release of DOX mediated by the photothermal effect was also studied at acidic pH. When irradiated with the NIR laser light for 3 min, the FA–NGO–PVP–DOX composite released more than 70% DOX in 10 h. The FA–NGO–PVP/DOX composite showed a dose-dependent effect of DOX on the viability of HeLa cells. At a lower concentration of DOX (2 µg/mL), the cell inhibition rate of the FA–NGO–PVP/DOX conjugate system was lower (18%) than the free DOX (27%) due to delayed DOX release from the FA–NGO–PVP composite. However, at a higher concentration of 20 µg/mL, in the presence of the NIR irradiation, the cell inhibition rate with the FA–NGO–PVP/DOX conjugate system was higher (90%) than the free DOX and FA–NGO–PVP/DOX, which were both about 70% [164].

**Figure 9:**
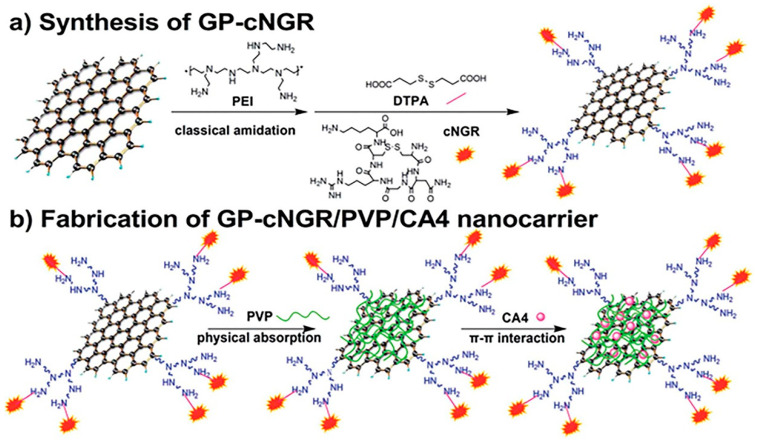
Schematic representation of the synthesis of cyclic aminopeptidase N (APN)-targeting peptide (cNGR)-modified functionalized graphene oxide (GO) nanosheets and preparation of drug delivery system GP–cNGR/polyvinylpyrrolidone (PVP)/Combretastatin A4 (CA4) [165]. Top: scheme (**a**); bottom: scheme (**b**). (Reprinted with permission from Ding et al. ‘A tumor-targeting drug delivery system based on cyclic NGR-modified, combretastatin A4-loaded, functionalized graphene oxide nanosheets’. *RSC Adv.* **2016**, *6*, 68134–68140. Copyright (2020) Royal Society of Chemistry).

Ding et al. designed a carrier based on aminopeptidase N (APN)-targeting peptide (NGR) functionalization of PVP-coated GO (Figure 9) [165]. At first, GO was prepared using modified Hummer’s method. The GO nanosheets were covalently modified with PEI to get the PEI-functionalized GO (GP) using EDC chemistry. Next, a 3,3-dithiodipropionic acid (DTPA) linker was attached to the amino groups on the GP surface, followed by the reaction of the cyclic NGR (cNGR) peptides and the carboxylic groups of DTPA to obtain the desired GP–cNGR. Subsequently, PVP was non-covalently loaded onto GP–cNGR through physical absorption. TEM images of pristine GO showed planar conformation and transparent GO sheets with some wrinkles. After functionalization with PVP, the size of GO reduced from several hundred nanometers to 200 nm, and the zeta potential of the composite decreased from −25.7 to −31.6 mV. The authors loaded anticancer drug Combretastatin A4 (CA4) via π–π stacking interaction to evaluate the nano-drug carrier efficiency of the GP–cNGR/PVP system. The resultant GP–cNGR/PVP/CA4 conjugate possessed high loading efficiency of 56%. At pH 7.4, 53.7% of the drug was released in 24 h in a sustained manner. An in vitro cytotoxicity study revealed that CA4 at 10 ng/mL concentration caused 62.4% and 45% inhibition of viability in HT-1080 and MCF-7 cells, respectively [165].

In another study, Karki et al. reported β-cyclodextrin and PVP-functionalized GO nanocarrier for delivery of water-insoluble anticancer drug SN-38 (7-ethyl-10-hydroxy camptothecin) in MCF-7 cells [166]. The authors synthesized PVP-modified GO through the covalent reaction of GO and PVP in the presence of catalysts 4-(Dimethylamino)pyridine (DMAP) and N,N′-dicyclohexylcarbodiimide (DCC). TEM images revealed density variations in peripheral and central regions of PVP-modified GO sheets. Further, the smooth surface of GO became coarse after surface modification and thickness of sheets increased after PVP functionalization. UV–VIS spectra of GO did not produce a sharp peak, but only a hump around 320 nm. However, GO–PVP displayed a sharp peak at 295 nm. The authors loaded SN-38, the active metabolite of anticancer drug irinotecan. The drug loading ratio was 17 wt%. At neutral, acidic, and alkaline conditions, about 13%, 30%, and 40% of the drug were released at 72 h, respectively. The GO–PVP carrier by itself showed no significant cytotoxicity against MCF cells in an MTT assay. Further, the GO–PVP–SN-38 system was significantly more cytotoxic (IC_50_ of 97 µM) than the free-drug SN-38 (IC_50_ = 288 µM) [166].

Recently, Tiwari et al. investigated the role of PVP-functionalized GO nanocarrier (GO–PVP) for subsequent delivery of two anticancer drugs, quercetin (QSR) and Gef, to ovarian cancer cell lines [167]. The authors first synthesized GO from expanded graphite powder using the modified Hummer’s method. PVP-functionalized GO was synthesized using a carbodiimide-activated esterification reaction. TEM images of GO sheets suggested endogenous wrinkled lamellar morphology. Interestingly, the GO–PVP surface appeared to be smooth. However, dark patches on PVP-modified GO sheets were observed. DLS studies revealed that the mean hydrodynamic diameter of GO was 166.5 nm, which became increased to 300–400 nm after surface functionalization with PVP. Zeta potential of GO-PVP was −50 mV, which suggests the stability of particles in solution or dispersion. The absorbance spectra of GO did not show any distinctive peak. However, PVP-functionalized GO displayed a characteristic peak around 290 nm. Both QSR and Gef were mixed sequentially with GO–PVP for drug loading, which turned out to be 20% for QSR and 46% for Gef. At pH 7.4, the release rate of drugs when combined was about 37% in 72 h. However, when used individually, the release rate was 20% and 18% for Gef and QSR, respectively. An in vitro cytotoxicity assay revealed no significant cytotoxicity towards normal human ovarian surface epithelial cell lines (IOSE). Further, the GO–PVP/QSR–GEF system showed 57% of inhibition of cell viability [167].

In a related study, Huang et al. reported a design of another effective photothermal therapeutic system [163]. PVP-functionalized rGO provides anchoring sites for a cyclic Arginine-Glycine-Aspartic Acid peptide (RGD4C). The rGO–PVP conjugate was synthesized by mixing GO, PVP, and L-ascorbic acid through pyrrolidine ring-opening and ester bond formation reaction. Further, RGD4C peptide was attached by mixing rGO–PVP and the peptide in PBS buffer at 70 °C. AFM studies revealed that the thickness of rGO–PVP increased from 0.85 to 2.81 nm after surface modification. Both rGO–PVP and rGO–PVP–RGD4C conjugate system displayed a sharp UV–VIS absorbance peak, peak maxima at around 300 nm. The resultant rGO–PVP–RGD peptide conjugate system was loaded with an aromatic photosensitizer Ce6, and this delivery system exhibited improved PDT towards the human gastric cancer cell line MGC 803 when compared with the thee free Ce6 alone [163]. We have outlined the GO–PVP composites in Table 4.

#### 4.1.8. Dextran-Functionalized GO Nanoparticles

Dextran (Dex) is a polysaccharide synthesized from the condensation of glucose. It is widely used in the medical field and in nanoparticle drug delivery and nanoparticle synthesis [168].

**Figure 10:**
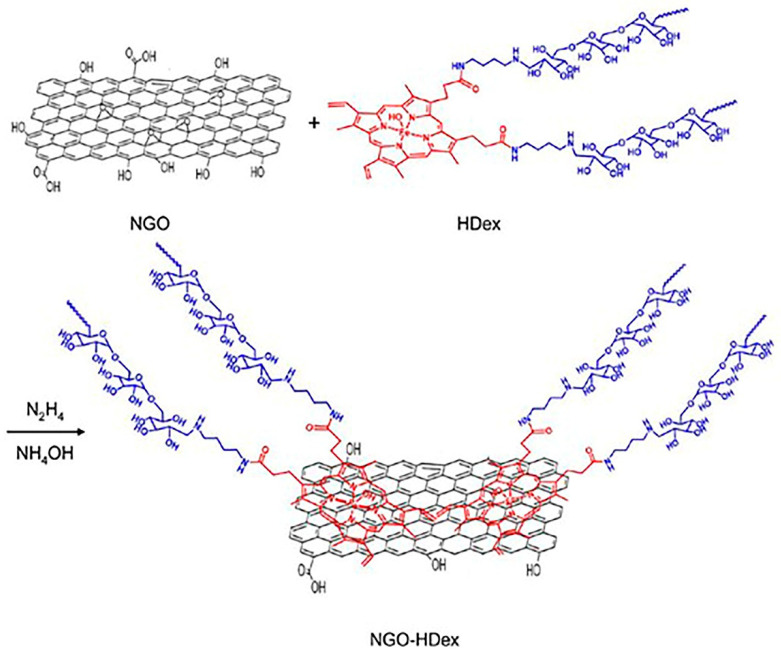
Synthesis of nanographene oxide (NGO)−dextran–hematin (HDex) hybrids [169]. (Reprinted with permission from Jin et al. ‘Self-assembled graphene-dextran nanohybrid for killing drug-resistant cancer cells’. *ACS Appl. Mater. Interfaces* **2013**, *5*, 7181–7189. Copyright (2020) American Chemical Society).

Jin et al. reported the synthesis of aminated Dex–hematin (HDex)-functionalized GO (HDex–NGO) nano-drug vehicle (Figure 10) in which HDex was conjugated to NGO through π–π stacking interactions [169]. Dex–NH_2_ was first synthesized by reacting Dex with N-boc-1,4-diaminobutane and sodium cyanoborohydride, followed by Boc removal using trifluoroacetic acid. Next, HDex was synthesized by coupling Dex–NH_2_ and hematin using EDC and NHS. Finally, HDex and NGO solutions were reacted in the presence of ammonia and hydrazine to obtain the NGO–HDex. UV–VIS spectra of NGO displayed a peak maximum at 230 nm and a hump at 300 nm, corresponding to π–π transitions of double bonds present in GO and n–π transition of carbonyl surface groups on GO. The UV–VIS peak at 230 nm in GO sheets was red-shifted to 260 nm in HDex–NGO. The hematin residue peak maxima at 386 nm was red-shifted in the HDex–NGO conjugate to 412 nm. All these peak shifts in UV–VIS absorbance spectra can be attributed to π–π interaction between NGO and HDex. DLS studies suggested that the mean hydrodynamic diameter of NGO–HDex increased from 178 nm in NGO to 220–240 nm in NGO–HDex. Zeta potential of NGO–HDex was found to be −11.7 mV, whereas the zeta potential of NGO was only −23 mV. The decrease in surface charge in NGO–HDex improved its stability in biological medium. The stability of the HDex–NGO conjugate was found to be better than the free NGO only. The authors successfully loaded DOX onto this HDex–NGO nano-drug vehicle with an excellent drug loading capacity of 3.4 mg/mg NGO using 0.3 mg/mL of DOX. The sustained release profile indicated that at pH 7.4, 11% DOX was released from this vehicle within 2 days. The authors reasoned that slow release could result in reduced toxicity to healthy cells. The HDex–NGO/DOX drug delivery system also exhibited some pH-dependent release as DOX released increased from 20% to 28% when pH was decreased from 7.4 to 5.5. The HDex–NGO itself was not significantly toxic to the adriamycin-resistant human breast cancer cell line (MCF-7/ADR) as the viability was more than 80% in the cell counting kit 8 (CCK-8) in vitro cytotoxicity assay. However, with the drug-loaded HDex–NGO/DOX system, cell viability was only 30%, even at a very low DOX (2 µg/mL) concentration. The cell viability using the same level of the free drug was 60%. The excellent drug loading capacity, slow and continuous drug release profile, and significant cytotoxicity of the drug-loaded HDex–NGO/DOX against the MCF-7/ADR cell line suggested chemotherapeutic efficiency of this nano-drug carrier system [169].

Xie et al. reported the non-covalent functionalization of GO with Dex and CS [124]. This GO–CS/Dex combination was used as a drug delivery vehicle for DOX. First, CS modification was performed by reacting GO and CS at pH 6.5. Next, Dex was reacted to GO/CS to obtain the GO–CS–Dex composite. DLS studies showed that the mean size of GO sheets was 263.86 nm, whereas GO–CS/Dex was 373.15 nm. AFM studies suggested that the thickness of GO sheets increased from 2 nm to 8–10 nm after surface functionalization with CS and Dex. Zeta potential studies suggested that surface charge of GO, GO–CS, and GO–CS–Dex were −33 mV, 28.89 mV, and −24.82 mV, respectively, suggesting layer by layer deposition of CS and Dex onto the GO sheets. DOX was loaded onto GO–CS/Dex through π–π stacking and electrostatic attractions. The authors reported a drug loading ratio of 64% with GO–CS–Dex composite. The functionalization with CS and Dex enhanced the dispersibility of both the GO and DOX-loaded GO nanosheets as no agglomeration was observed under physiological conditions. The functionalized GO also provided the benefit of reduced non-specific protein adsorption. The pH-dependent drug release from the GO–CS/Dex/DOX composite was evident, as, at pH 7.4, 28.9% DOX was released in 120 h as compared to 49% release at pH 5.0. MTT assay suggested lower cytotoxicity of the GO–CS/Dex system. Further, the GO–CS/Dex/DOX system was found to be cytotoxic to MCF-7 and HepG2 cells [124].

Hu et al. designed a self-assembled rGO–Dex system through non-covalent H-bonding interactions [170]. Dex-modified rGO was synthesized by reacting GO and Dex in ultrasound conditions in the presence of ammonium hydroxide. GO showed an absorption peak at 229 nm, while rGO–Dex showed peak maxima at 213 nm and 268 nm. DLS studies suggested the hydrodynamic sizes to be 32.7 nm, 34.6 nm, 42.9 nm, 44.3 nm, and 150 nm at days 1, 3, 5, 7, and 10, respectively, which suggested they did not form aggregate aqueous solution in a short time. TEM images showed transparent GO sheets with some wrinkles of size more than 500 nm; the size of rGO–Dex decreased to 60 nm. DOX was loaded onto rGO–Dex composite for photo-chemotherapy. Further, oligopeptide RGD was introduced onto the composite system for better targeted therapeutic efficiency. The rGO–Dex/DOX and rGO–Dex–RGD/DOX system that combined both chemotherapeutic and NIR photothermal ablation was found to be more effective than single chemotherapy against the murine mouse melanoma B16F10 cell line [170].

In another study, Zhang et al. designed a layer-by-layer self-assembled GO/FeO/CS/Dex nanocarrier system (GO-IONP-CS-Dex) for magnetically targeted drug delivery and photothermal ablation [171]. GO sheets were dispersed in ethylene glycol-diethylene glycol medium, and FeCl_3_·6H_2_O was reacted to GO in the presence of sodium acetate and sodium acrylate to make GO–IONP composite in the solvothermal method. Next, CS and Dex were added sequentially to the GO–IONP composite. TEM images of GO–IONP composite suggested that 5–10 nm-sized IONP particles were deposited onto GO sheets. GO–IONP composite exhibited strong magnetic properties that were not affected after surface functionalization with CS and Dex. AFM studies revealed that the thickness of GO, GO–IONP, GO–IONP–CS, and GO–IONP–CS-Dex were 1, 2, 6–9, and 40–50 nm, while the zeta potentials were −33.82, −29.36, 40, and −35 mV, respectively. DOX was loaded onto the GO/FeO/CS/Dex system through π–π stacking and electrostatic attractions, with a loading ratio of 140.4 wt%. The release of DOX from GO/FeO/CS/Dex at pH 7.4 and 5.0 was 36.5% and 59.2% at 168 h and 72 h, respectively. MTT assays suggested no apparent cytotoxicity of the GO/FeO/CS/Dex system to human lung cancer A549 cells. The cytotoxicity of GO/FeO/CS/Dex/DOX was higher compared to the GO–FeO/DOX system. Further, the GO/FeO/CS/Dex system also exhibited a good photothermal therapeutic effect against A549 cells upon exposure of 808 nm NIR laser irradiation at a power density of 1 W/cm^2^ [171].

Kiew et al. reported GO100-Dex composites as a nanocarrier for the delivery of DOX to human umbilical vein endothelial cells (HUVECs) [172]. Dex-conjugated GO (GO100-Dex) was synthesized through a modified esterification reaction of GO100 (nano-sized GO obtained through ultrasonication of GO sheets) and Dex in the presence of EDC as a coupling agent. The GO100–Dex size was determined to be 100–200 nm. Zeta potential measurements suggested surface charge of nano GO sheets changed from −55 mV to −44 mV after surface modification. GO showed absorption maxima at 230 nm and a shoulder peak at 300 nm. Surface modified GO showed similar absorption spectra with a slight increase in absorbance in 300–600 nm. Drug loading capacity using this nanocarrier was quite high at 64%. The release of DOX was 28% at pH 7.4, which increased to 48% at an acidic pH 5.8. Further, cytotoxicity studies suggested that at pH 7.4, free DOX was more toxic than GO100–Dex/DOX. However, at pH 6.6, in the presence of α-amylase, simulating tumor conditions, GO100–Dex/DOX was far more toxic than free DOX [172]. We have listed the GO-Dex composites in Table 5.

### 4.2. GO Antibody Nanocomposites

Several antibodies have found use in GO-based nano-drug carrier systems for cancer therapy. Zhou et al. designed a pH-responsive integrin αvβ3 monoclonal antibody and charge reversal polyelectrolyte-modified GO nanocomposites for specific targeted delivery of DOX, both in vivo and in vitro [173]. The cationic PEI was coated onto GO, followed by the attachment of the integrin αvβ3 monoclonal antibody to the carboxylic acid groups of GO through an amide-coupling reaction. Next, a charge reversal polyelectrolyte (PAH-Cit) was loaded onto PEI-coated GO. Finally, DOX was loaded onto GO by covalent attachment to PAH-Cit to obtain a self-assembled system GO–Abs/PEI/PAH-Cit/DOX nanocarrier. The loading capacity increased linearly with increasing DOX concentration, reaching a maximum of 0.294 mg/mg of the carrier with 0.32 mg/mL of initial DOX concentration, suggesting a loading efficiency of 92%. At pH 7.4, ≈28% of the drug was released from the nanocarrier in 24 h, whereas the release of drugs was 50% and 81% at pH 6.8 and pH 5, respectively. U87 MG human malignant gliomas cell line-overexpressing integrin αvβ3 antibody was used to evaluate the cytotoxic effect of the GO–Abs/PEI/PAH-Cit/DOX nanocarrier system. The CCK-6 assay suggested that the GO–Abs/PEI/PAH-Cit/DOX was more cytotoxic to U87 MG cells compared with the carrier without the integrin αvβ3-targeting antibody [173].

Zheng et al. reported the design of anti-HER2-antibody conjugated poly-L-lysine-coated rGO (anti-HER2–rGO–PLL) nano-drug vehicle for delivery of DOX against the HER2-overexpressing MCF-7 cells (MCF7/HER2). DOX was loaded onto the anti-HER2–rGO–PLL nanocarrier via π–π stacking and hydrophobic interactions with a drug-loading ratio of 37.2%. The release of DOX was deficient even after 3 days, which suggests that the anticancer drug could be protected in the bloodstream before reaching out to specific targeted cancerous cells. The anti-HER2–rGO–PLL/DOX and rGO–PLL/DOX showed cytotoxic IC_50_ values of 0.8 µg/mL and 6 µg/mL in MCF7/HER2 cells, respectively, indicating improved anticancer efficacy with the antibody-targeted nanocarrier [174].

Recently, Bugárová et al. reported a GO platform decorated with iron-based magnetic nanoparticles (MNps) and monoclonal antibody M75 for the CA IX receptor. Two types of nanoplatforms were fabricated, GO–MNps–EDC–MAb and GO–MNps–Mab, with and without the coupling agent EDC. Both GO–MNps–EDC–MAb and GO–MNps–Mab were nontoxic to B16 neo cells but showed a slight cytostatic effect towards mouse melanoma cells B16-F0 expressing CA IX [175].

Wei et al. fabricated a multifunctional NGO-based PDT and drug delivery system with phototoxicity on–off properties. Pyrophephorbide-a (PPa) was used as a photosensitizer, and integrin αvβ3 monoclonal antibody (mAb) was used for targeted tumor therapy. PEG was introduced in the system for better aqueous solubility and stability. The authors fabricated the PPa–PEG–NGO–mAb conjugate system for tumor targeting and PDT. The authors demonstrated that the PPa–PEG–NGO–mAb conjugate system was capable of selectively targeting the cancerous cells overexpressing integrin αvβ3. After internalization into tumor cells, this nanocarrier system escapes lysosomes and is transferred to mitochondria, where, in the ON state, it effectively causes phototoxicity to tumor cells [176].

Tran et al. investigated another GO-based platform, in which mesoporous silica nanoparticles (MSNs) were decorated internally with fluorescent conjugates and externally with polydopamine (PDA) and GO layers. Further, the MSNs were conjugated with a monoclonal anti-human EGFR antibody for targeted delivery of cisplatin. This GO–PDA–MSN–Ab nano-drug delivery vehicle gave pH- and NIR-dependent controlled drug release. The multifunctional carrier showed low cytotoxicity towards healthy HEK293 cells, but when loaded with cisplatin, the nanocarrier showed high cytotoxicity to human neuroblastoma SH-SY5Y cells [177].

### 4.3. GO–Metal Nanoparticle Composites

Recently, a few research groups have reported the anticancer effect of GO–metal nanoparticle composites by themselves without any chemotherapeutic agent. Gurunathan et al. fabricated an rGO–AgNP composite using *Tilia amurensis* plant extract and explored its anticancer potential in ovarian cancer cells (A2780) [178]. The synthesized rGO–AgNP nanocomposites were highly stable and water-soluble and did not aggregate for 3 months. The rGO–AgNP composite exhibited a dose-dependent inhibition of viability with an IC_50_ value of ≈12.5 µg/mL. The composite resulted in the loss of cell membrane integrity, as evidenced by enhanced lactate dehydrogenase leakage. Further, the rGO–AgNP system increased ROS generation and DNA fragmentation in A2780 cells, demonstrating its potential in cancer treatment [178].

Kavinkumar et al. [179] synthesized GO/rGO–AgNP nanocomposites and explored the anticancer effect of this conjugate system against the human lung cancer A549 cell line. The authors first synthesized GO and rGO following conventional Hummer’s method, and Ag nanoparticles were synthesized using traditional methods using vitamin C as a reducing agent. Finally, negatively charged Ag nanoparticles were adsorbed onto the surface-modified positively charged GO/rGO via electrostatic attractions. The cytotoxicity of GO/AgNPs, rGO/AgNPs, and GO were evaluated against A549 cells by MTT assay. For GO only, even at a very high concentration (200 µg/mL) after 24 h, the cell viability was higher than 40%. This low cytotoxicity of GO on lung cancer cells can be due to oxygen-containing functional groups, e.g., OH, -COOH, and epoxy groups on the GO surface [180]. Cytotoxicity of rGO was slightly higher than GO, with cell viability IC_50_ values of 160 µg/mL and 180 µg/mL, respectively, after 24 h. Further, the rGO–AgNP nanohybrid system demonstrated better anticancer activity (IC_50_ of 30 µg/mL) than the GO–AgNP composite (IC_50_ of 100 µg/mL) against the A549 cell line. The authors suggested that the improved anticancer activity of the rGO–AgNP composite was a result of the synergistic effect of rGO and AgNPs and enhanced intracellular delivery of rGO. GO–AuNP nanocomposites have been well studied for cancer therapy [181,182,183,184,185].

More recently, Lina et al. reported the synthesis of rGO–curcumin (CUR)-capped gold (CAG) nanoparticle composite and investigated its efficiency as an antioxidant and anticancer agent [186]. CAG was synthesized using reducing properties of CUR and, alternatively, following conventional sodium citrate reduction as well. The activity of CUR-capped AuNP–rGO composite was tested against two human colon cancer cell lines, namely, HT-29 (colon adenocarcinoma) and SW948 (Duke’s C colorectal carcinoma). Cytotoxicity studies revealed that both cancer cells displayed a change in size and morphology upon CAG treatment compared to the control. Optical microscopy revealed cellular shrinkage and inhibition of proliferation in a dose- and time-dependent manner. The cytotoxic effect of CAG in the WST-8 assay showed a cell viability IC_50_ of 100 µg/mL in HT-29 and SW-948, cells, while only very low inhibition to normal colon cell line (CCD-841) was observed. This composite also showed low inhibition in normal liver cells (WRL-68) as a RES organ. The 2,2-diphenylpicrylhydrazyl (DPPH) assay showed CAG’s ability to inhibit free radicals and exert antioxidant effects to neutralize reactive oxygen species (ROS) and inflammatory intracellular tumor microenvironments [186].

Thapa et al. fabricated a methotrexate (MTX)-functionalized GO (MTX/GO)–AgNP composite for folate receptor-targeted cancer therapy [187]. They investigated the effect of MTX–GO–AgNP nanocomposites against folate receptor-expressing MCF-7 cells and against HepG2 cells that do not express the folate receptor. The MTX–GO/AgNP composite system demonstrated enhanced uptake and greater cytotoxicity towards MCF-7 cells than the HepG2 cells. Further, due to the presence of GO, the MTX–GO–AgNP composite exhibited a pronounced NIR laser-induced photothermal effect on tumor cells. Moreover, MTX–GO–AgNP composite increased ROS levels, leading to enhanced apoptosis [187].

Kang et al. fabricated GO–AuNP nanocomposites and explored its potential for PTT [188]. The GO/AuNP hybrid system was synthesized by sandwiching GO sheets between the two layers of α-synuclein-coated AuNPs. The GO/AuNP sheets can be loaded to the tumor-tropic mesenchymal stem cell (MSC) surface for enhanced photothermal efficiency for cancer treatment [188].

In another study, Maji et al. reported the design of a nanohybrid system comprising griseofulvin (GSF)-coated AuNP–mesoporous silica-coated rGO conjugated with FA (GSF–AuNP–mSi NP–rGO) and investigated its potential as an artificial enzyme for in vitro cancer detection and cancer therapy [189]. The GSF–AuNP–mSi NP–rGO nanocomposites showed peroxidase-like activity and was used as a quantitative and colorimetric detection probe for cancer cells. Further, the nanocomposites were not toxic to normal human embryonic kidney (HEK) 293 cells but resulted in an increase in ROS and cytotoxicity to HeLa cells. Thus, this GSF–AuNP–mSi NP–rGO nanohybrid system demonstrated its ability to detect and selectively kill the tumor cells [189].

## 5. In Vivo Biocompatability of Graphene-Based Nanoparticles

In vivo biocompatibility of GBNs has been reported in a dose- and time-dependent toxicological evaluation in animal models. We have briefly summarized below the biocompatibility of functionalized and unfunctionalized GBNs. More detailed reviews focused on this topic can be found in the literature [190,191]. Zang et al. reported the application of AgInZnS–graphene oxide (GO) nanocomposites for in vivo imaging in SK-BR-3 breast cancer cells of tumor-bearing mice [192]. The nanocomposite was determined to be around 100 nm in size and 10 nm in height. In vivo imaging in nude mice bearing breast tumors revealed that the nanocomposites were widely distributed in tumor tissues. Fluorescence imaging in harvested organs suggested that the particles were mostly found in the heart, liver, lung, spleen, and kidneys [192]. Shi et al. reported in vivo biodistribution behavior of CUR with unfractionated heparin-functionalized rGO (UFH–rGO) [193]. The nanocomposites have a size of less than 100 nm. It was noted that after 4 or 8 h of administration, the concentration of free CUR in mice liver and other major tissues was lower than that achieved through the rGO–UFH/CUR composite. There was no distribution of particles in mice brain. Further, in vivo cytotoxicity and biocompatibility suggested that rGO–UFH/CUR had good biocompatibility and negligible cytotoxicity to mice heart, liver, spleen, lungs, and kidney.

Very recently, Vuppaladadium et al. reported better biocompatibility of silanized GO [194]. The silanized GO sheets (SiGO) had multi-layered morphology with sizes in the micrometer range. In vivo toxicity studies revealed no reduction in body weight or decrease in the relative weight of liver, kidney, spleen, and lungs of silanized GO-treated mice vs. the control group. Further, histopathological sections suggested similar histology of all of the organs, and kidney sections suggested clean glomeruli of both the control and SiGO-treated mice.

Deng et al. reported applications of human serum albumin-functionalized PEG-coated GO nanosheets for the delivery of PTX [195]. The nanocomposite had a size of 191 nm. In vivo cytotoxicity studies suggested that the functionalized GO nanocarrier did not exhibit any cytotoxicity to major organs such as the heart, liver, spleen, lungs, and kidneys of mice after 1 month of treatment. In another study, Yang et al. studied the in vivo toxicity, pharmacokinetics, and long-term biodistribution of ^125^I radionuclide-labeled and PEG functionalized nanographene sheets [196]. The authors reported that the PEGylated NGO, of 10–30 nm in size, after i.v. administration in Balb/c mice at 4 mg/kg dose accumulated in the RES, but could be gradually cleared by both renal and hepatic excretion. Further, PEGylated NGO when given at 20 mg/kg did not cause appreciable toxicity in mice over 3 months. In another study, acute and chronic toxicity of few-layer graphene and its carboxylated and PEG-functionalized derivatives was evaluated after i.v. administration (20 mg kg^−1^) in Swiss albino mice over 3 months [197]. The authors found that while the few-layer pristine graphene of lateral dimension of ≈100–200 nm or its carboxylated derivative showed significant toxicity, the PEGylated derivative, despite accumulation, did not induce any noticeable toxicity or damage to the lung, liver, kidney, or spleen.

Zhang et al. evaluated in vitro cytotoxicity and in vivo biocompatibility of Dex-functionalized grapheme [198]. The authors noted that GO–DEX of lateral size 50–100 nm upon i.v. administration in mice accumulated in the RES and was cleared within 1 week without causing noticeable short-term toxicity. Kanakia et al. reported the sub-acute toxicity of an MRI-contrasting agent comprising manganese-intercalated and Dex-functionalized graphene nanoparticles (Mangradex) [199]. Intravenous administration of 1, 50, and 100 mg/kg dosages of Mangradex of size 100 nm three times/week for 3 weeks in rodents did not induce an inflammatory response in major organs, including the brain, heart, liver, lung, kidney, and spleen, and further caused no noticeable changes in hematological parameters.

Zhang et al. demonstrated the distribution and biocompatibility of GO in mice [200]. The authors showed that GO of lateral width 10–800 nm gets predominantly deposited in the lungs, but when compared to other carbon nanomaterials, GO showed low uptake in the RES. Further, at 1 mg·kg^−1^, bodyweight GO did not cause any pathological changes in lung, liver, kidney, and spleen for 14 days. Further, a larger dosage of 10 mg·kg^−1^ body weight also did not change the pathophysiology of all organs except for the lungs, where the accumulation of GO resulted in inflammatory cell infiltration, pulmonary edema, and granuloma formation.

Yan et al. reported intraocular biocompatibility and cytotoxicity of GO both in vitro and in vivo [201]. The authors showed that GO did not cause any significant changes in cell viability and proliferation. The authors demonstrated that GO sheets of 1 nm thickness had good intraocular biocompatibility. When intravitreally injected in rabbit eyes at doses of 0.1–0.3 mg, GO did not cause significant changes in the eyeball appearance, intraocular pressure, electroretinogram, and histological examination. In another study, Ali-Boucetta et al. compared the effect of conventional GO with highly pure, single layer, colloidally stable, and dispersed GO nanoparticles of lateral dimension less than 500 nm. The authors found that the purified GO does not induce any significant cytotoxicity in vitro or inflammation or granuloma upon intraperitoneal injection [202].

Liu et al. studied the effect of size and dose (1.0–10 mg/kg of body weight) on the biodistribution of GO in mice [203]. The large size GO was 1–5 µm while the small size GO was 100–500 nm in size. The authors found that, regardless of size, GO was cleared from the blood quickly and accumulated mainly in the liver and lungs. The study revealed that increasing injecting dose and GO particle size causes higher accumulation in the lungs. On the other hand, small size GO is mainly accumulated in the liver [203]. In another study, Duch et al. evaluated the biocompatibility of graphene nanomaterials in the lung and demonstrated that the oxidation of graphene to GO (of size 0.5 to 2.0 nm) is a significant contributor to pulmonary toxicity when injected directly to the lungs of mice [204]. On the other hand, the toxicity was significantly reduced with pristine graphene through liquid-phase exfoliation or when nanoscale dispersion of graphene was dispersed in nonionic amphiphilic block copolymer Pluronic. Singh et al. reported that single- or few-layer GO sheets (0.2 to 5 μm) caused strong platelet aggregation through activation of Src kinases [205]. The authors noted that i.v. administration of GO induced pulmonary thromboembolism in mice, while rGO was significantly less effective in aggregating platelets, suggesting the role of surface charge distribution in platelet aggregation [205]. In another study, Wang et al. compared the immune response resulting from the i.v. administration of graphene nanosheets and multiwalled carbon nanotubes in C57BL/6 mice [206]. The authors demonstrated that the use of graphene nanosheets or multiwalled carbon nanotubes of size 2–25 nm can induce site-specific Th2 inflammatory responses via the IL-33/ST2 axis.

Wen et al. studied the long-term biodistribution and toxicity of i.v. NGO functionalized with poly sodium 4-styrenesulfonate (NGO-PSS) over 6 months in mice [207]. When given in a higher dose of 18 mg/kg, the PSS-functionalized NGO, with large lateral size of around 500 nm, accumulated in the liver, lung, and spleen, causing acute liver injury and chronic inflammation of these organs. Zhang et al. looked into the short- and long-term effects of the exposure of orally administered high dose rGO nanosheets (87–472 nm size) on the learning and memory behavior of mice [208]. The authors found that although the general locomotor activity, balance, and neuromuscular coordination were initially affected, the mice showed little change in anxiety-like or learning and memory behaviors. In another study, Wong et al. evaluated GO’s biocompatibility by studying its effect on human fibroblast cells and in vivo in mice [209]. A total of 35 mice were divided into three test groups that received 0.1, 0.25, and 0.4 mg of GO of 1 nm in size. The histological analysis was carried out after 1, 7, and 30 days, respectively. No apparent toxicity to mice was observed from the lower and the middle doses; however, the high dose (0.4 mg) exhibited chronic toxicity resulting in the death of four out of nine mice.

## 6. Limitations and Challenges of GBNs

Although GO is increasingly used for in vivo applications (Table 6), there is a concern associated with the use of GBNs because of their accumulation in organs and the potential to cause short- and long-term toxicity upon acute or chronic exposure. The immunotoxicological heterogeneity and hemolytic potential and toxicity associated with GBNs may result from the different techniques involved in its preparation and reagents used in its synthesis, such as surfactants, coating, and reducing agents, as well as metal impurities that potentially can contaminate the final product [209]. GBN organ biodistribution is influenced by their size, lateral dimensions, number of layers, C/O ratio, surface properties, and surface functionalization. GBNs are toxic to prokaryotic cells and have been found to be useful as antibacterial agents [210]. Cytotoxicity and in vivo biocompatibility studies of GBNs suggest that while graphene and GO can accumulate and can cause potential toxicity, appropriate functionalization of pristine or as-made GO can avoid much of its toxic effect, and functionalized GO holds promise as a nanocarrier for drug delivery, imaging, and photothermal therapy.

## 7. Conclusions and Future Perspectives

Over the past few decades, there have been significant advances in nanomaterial usage for biomedical applications. Of late, GO has emerged as a promising material in nanomedicine. Surface functionalization of GO plays a vital role in reducing toxicity and increasing the stability, biocompatibility, and solubility of GO in the physiological buffer. Surface-functionalized GO has become a popular nanocarrier, specifically for targeted drug delivery systems and cancer therapy. GO absorbs in the NIR region and GO–metal nanoparticle nanocomposites are efficient in cancer cell inhibition and have application in PTT. GO is also fabricated as a carrier for PS and is shown to increase the selectivity and efficacy of PDT. Most of the currently approved nanodrugs rely on EPR and are more effective in improving bioavailability and pharmacokinetics of the drug than enhancing the efficacy of the chemotherapeutic agent. However, many next-generation nanodrugs in clinical trials are now based on active targeting and use stimuli-responsive and multifunctional nanomaterials. Some of these nanocarriers may overcome current challenges and are expected to provide new treatment options in fighting unmet medical needs in cancer therapy and beyond.

## Figures and Tables

**Table 1:** The graphene oxide (GO)–chitosan (CS) composites developed, the drug used, the type of cancer and cell treated, and the drug loading and release efficiency.

GO–CS Nanocomposites	Size	Drug Used	Cancer Cell Line	Drug Loading Efficiency	Drug Release Efficiency	Reference
rGO–CS–TPP	340.55 ± 21.78 nm	DOX	Prostate cancer cells (PC-3)	65%	50% in 48 h	[123]
GO–CS–Dex	263.86 ± 5.9 nm	DOX	Human breast cancer cells (MCF-7)	63.7%	28.9% at pH 7.4, 49.1% at pH 5.0	[124]
GO–CS-γ–PGA	200–300 nm	DOX	Human cervical cancer cells (HeLa)	118.83%	5.37% at pH 7.4, 52.58% at pH 5	[121]
GO–CS–CS/DMMA	114 nm	DOX	Human liver cancer cells (HepG2)	89.35% ± 4.32%	5.1% at pH 7.4, 56.4% at pH 5 in 6 h, 84.75% in 18 h	[131]
GO–CS	several hundred nanometers to several micrometers	IBU	Human lymphoblastic leukemia cells (CEM) and human breast cancer cells (MCF-7)	9.7%	10% at pH 1.4, 19% at pH 7.4	[127]
		5-FU		5.3%	70% at pH 1.4, 50% at pH 7.4	

**Table 2:** Graphene oxide (GO)–polyethylene glycol (PEG) composites, the drug used, type of cancer cell treated, drug loading, and release efficiency.

GO–PEG Nanocomposites	Size	Drug Used	Cancer Cell Line	Drug Loading Efficiency	Drug Release Efficiency	Reference
GO–IONP–PEG	50–300 nm	DOX	Murine breast cancer cells (4T1)	100%	20% at pH 7.4, 50% at pH 5	[44]
NGO–PEG NH_2_, later conjugated with antibody Rituxan for better targeting	≤20 nm	DOX	Hematopoietic human cancer Raji cells	not defined	15% at pH 7.4, 40% at pH 5.5	[120]
GO–PEG 4000	not defined	DOX	Adenocarcinomic human alveolar basal epithelial cells (A549)	87%	2.5% at pH 7.4 in 1 h, 3.5% at pH 5.8 in 1 h	[138]
NGO–PEG NH_2_	146.10 nm	Pt and DOX	Human tongue squamous carcinoma cells (CAL-27) and human breast cancer cells (MCF-7)	DOX: Pt: NGO–PEG = 0.376:0.376:1	30.0% (Pt) and 41.0% (DOX) in more than 72 h at pH 7.4, 65.7% (Pt) and 64.6% (DOX) at pH 5.3 in 72 h	[139]
FA–PEG–GO, later conjugated with peptide for targeted delivery	GO size 100 nm, size of FA–PEG–GO not defined	CPT	Human cervical carcinoma cells (HeLa)	90%	21.5% at pH 7.4, 71% at pH 5	[141]
GO–PEG NH_2_, later conjugated with photosensitizer Ce6 for synergistic cancer photodynamic therapy	≈170 nm	DOX	Mouse squamous carcinoma cells (SCC7)	51.9 ± 5.1% for Ce6 and 61.7 ± 4.4% for DOX	Not defined	[142]
GO–PEG NH_2_	50–200 nm	PTX	Adenocarcinomic human alveolar basal epithelial cells (A549) and human breast cancer cells (MCF-7)	11.2%	Not defined	[140]

**Table 3:** Graphene oxide (GO)– hyaluronic acid (HA) composites, their size, the drug used, type of cancer cell treated, drug loading, and release efficiency.

GO–HA Nanocomposites	Size	Drug Used	Cancer Cell Line	Drug Loading Efficiency	Drug Release Efficiency	Reference
GO–HA	40–350 nm	DOX	HeLa human cervical cancer cells (L-929)	81.5%	6.8% for pH 7.4, 10.9% for pH 6.3, 26% at pH 5.2	[154]
GO–HA	78.1 nm	Photosensitizer Ce6	Human cervical cancer cells (HeLa) cells and mouse embryonic fibroblast cells (NIH3T3)	115%	7% at pH 5, 22% at pH 7, and 30% at pH 9	[155]
GO–HA–RGD peptide	70–490 nm	DOX	Human ovarian cancer cells (SKOV-3)	72.9%	30.2% at pH 5.5, 7.6% at pH 7.4	[156]
NGO–SS–HA	125 nm	Gef	Adenocarcinomic human alveolar basal epithelial cells (A549)	13.8%	30.8% in absence of GSH, 60.1% in presence of GSH	[153]
NGO–HA	250 nm	Epirubicin	Murine melanoma cells (B16F1)	2% at pH 4, 9% at pH 7, 25% at pH 9	70% at pH 5, 18% at pH 7	[152]
GO–HA, later combined with iron oxide NPs for magnetic field-enabled chemotherapy for better cancer cell inhibition	166.8 ± 16.2 nm	DOX, PTX	Human breast cancer cells (MDA-MB-231)	33.5 ± 1.4%	53% at pH 7.4, 61% at pH 5.5	[158]
GO–HA	10−200 nm	DOX	Human liver cancer cells (HepG2)	42.9%	20% at pH 7.4, 40% at pH 5.3	[151]
rGO–HA-g-PMAO	108 nm	Used for PTT	MCF-7 human breast cancer cells	-	-	[157]

**Table 4:** The graphene oxide (GO)–Polyvinylpyrrolidone (PVP) composites developed, their size, the drug used, the type of cancer cell treated, drug loading, and release efficiency.

GO–PVP Nanocomposites	Size	Drug Used	Cancer Cell Line	Drug Loading Efficiency	Drug Release Efficiency	Reference
rGO–PVP–RGD		Photosensitizer Chlorin e6	Human gastric cancer cells (MGC 803)	7.41%		[163]
GO–cNGR/PVP	204.6 nm	CA4	Human fibrosarcoma cells (HT-1080) and human breast cancer cells (MCF-7)	56.3%	53.7% at pH 7.4	[165]
FA–NGO–PVP	<100 nm	DOX	Human cervical cancer cells (HeLa)	107.5%	60% at pH 5.5, 13% at pH 7.4	[164]
GO–PVP	Few hundred nm	SN-38	Human breast cancer cells (MCF-7)	17%	11–13% at pH 7, 26–30% at pH 5	[166]
GO–PVP	300–400 nm	QSR and Gef	Human ovarian teratocarcinoma cells (PA-1)	20% of QSR and 46% of GEF	34–37% at pH 5, 18–20% at pH 7	[167]

**Table 5:** The graphene oxide (GO)–dextran (Dex) composites, their size, the drug used, the type of cancer cell treated, drug loading, and release efficiency.

GO–Dextran Nanocomposites	Size	Drug Used	Cancer Cell Line	Drug Loading Efficiency	Drug Release Efficiency	Reference
GO–IONP–CS–Dex	425.33 ± 3.91 nm	DOX	Human lung cancer cells (A549)	140.4%	36.5% at pH 7.4, 59.2% at pH 5.0	[171]
NGO–HDex	223–239 nm	DOX	Multidrug-resistant breast cancer cells (MCF-7/ADR)	Above 90%	11% at pH 7.4, 28% at pH 5.5	[169]
rGO/DOX/RGD–Dex	60 nm	DOX	Murine melanoma cells (B16F10)	19.75%	7.4% at pH 7.4, 12.7% at pH 6.8, 38.4% at pH 5.3	[170]
GO–CS/Dex	373.15 ± 0.67 nm	DOX	Human breast cancer cells (MCF-7) and human liver cancer cells (HepG2)	63.7%	28.9% at pH 7, 49.1% at pH 5	[124]
GO_100_–Dex	133 ± 7.18 nm	DOX	human umbilical vein endothelial cells (HUVECs)	64%	48% at pH 5.8, 20% at pH 7	[172]

**Table 6:** In vivo studies on graphene-based nanomaterials (GBNs).

GBNs	Size	In Vivo Study	Reference
PEGylated GO–IONP	10 nm	Drug delivery/DOX for breast cancer	[44]
NGO–SS–HA composite	125 nm	Drug delivery/gefitinib for lung cancer	[153]
GO–adipic acid–HA conjugate	40–350 nm	Drug delivery/DOX for cervical cancer	[154]
AgInZnS–graphene oxide	100 nm	In vivo imaging in SK-BR-3 breast cancer cells of tumor-bearing mice	[192]
UFH–rGO	<100 nm	Biocompatibility study/good biocompatibility and negligible cytotoxicity to mice heart, liver, spleen, lungs, and kidney	[193]
Silanized GO	micrometer range	Biocompatibility study/no reduction in body weight or decrease in the relative weight of organs	[194]
Albumin-functionalized PEG-coated GO	191 nm	Biocompatibility study/no cytotoxicity to major organs such as heart, liver, spleen, lungs, and kidneys of mice after 1 month of treatment	[195]
PEGylated NGO	10–30 nm	Biocompatibility study/did not cause appreciable toxicity in mice over 3 months	[196]
Few-layer pristine graphene	100–200 nm	Biocompatibility study/PEGylated derivative despite accumulation did not induce any noticeable toxicity	[197]
GO–DEX	50–100 nm	Biocompatibility study/accumulated in the RES and got cleared within 1 week without causing noticeable short-term toxicity	[198]
Manganese and Dex-functionalized graphene nanoparticles	100 nm	Biocompatibility study/did not induce an inflammatory response in major organs	[199]
GO	10–800 nm	Biocompatibility study/did not change the pathophysiology of all organs except lungs	[200]
GO nanosheets	1 nm	Biocompatibility study/no significant changes in the eyeball appearance, intraocular pressure, electroretinogram, and histological examination was observed	[201]
GO	<500 nm	Biocompatibility study/purified GO did not induce inflammation or granuloma upon intraperitoneal injection	[202]
Large GO	Large GO: 1–5 µm, small GO: 100–500 nm	Biocompatibility study/increasing injecting dose and GO particle size caused higher accumulation in the lungs; on the other hand, small size GO was mainly accumulated in the liver	[203]
GO	0.5–2.0 nm	Biocompatibility study/GO (of size 0.5 to 2.0 nm) was a significant contributor to pulmonary toxicity when injected directly to the lungs of mice; the toxicity was significantly reduced with pristine graphene through liquid-phase exfoliation or dispersion of graphene	[204]
GO	0.2–5 μm	Biocompatibility study/i.v. administration of GO-induced pulmonary thromboembolism in mice, while rGO was significantly less effective in aggregating platelets	[205]
Graphene nanosheets or multiwalled carbon nanotubes	2–25 nm	Biocompatibility study/induced site-specific Th2 inflammatory responses via the IL-33/ST2 axis	[206]
NGO–PSS	500 nm	Biocompatibility study/accumulation in the liver, lung, and spleen, causing acute liver injury and chronic inflammation	[207]
reduced graphene oxide nanosheets	87–472 nm	Biocompatibility study/mice showed little change in anxiety-like or learning and memory behaviors	[208]
GO	1 nm	Biocompatibility study/high dose (0.4 mg) exhibited chronic toxicity resulting in the death of four out of nine mice	[209]

## Abbreviations

GO	Graphene oxide
rGO	Reduced graphene oxide
AgNPs	Silver nanoparticles
AuNPs	Gold nanoparticles
NIR	Near-infrared
PTT	Photothermal therapy
EPR	Enhanced permeability and retention
RES	Reticuloendothelial system
VEGF	Vascular endothelial growth factor
GBNs	Graphene-based nanoparticles
PDT	Photodynamic therapy
PEG	Polyethylene glycol
PEI	Polyethylenimine
PAA	Polyacrylic acid
PVA	Polyvinyl alcohol
NGO	Nanographene oxide
CS	Chitosan
FA	Folic acid
DOX	Doxorubicin
Dex	Dextran
PTX	Paclitaxel
5-FU	5-fluorouracil
IBU	Ibuprofen
CPT	Camptothecin
PVP	Polyvinylpyrrolidone
ROS	Reactive oxygen species
HA	Hyaluronic acid
NHS	N-hydroxysuccinimide
EDC	1-Ethyl-3-(3-dimethylaminopropyl)carbodiimide
AFM	Atomic force microscopy
MTT	3-(4,5-dimethylthiazol-2-yl)-2,5-diphenyl tetrazolium bromide
DLS	Dynamic light scattering
CUR	Curcumin
Gef	Gefitinib
XRD	X-ray diffraction
FTIR	Fourier transform-infrared spectroscopy
TGA	Thermo gravimetric analysis
XPS	X-ray photoelectron spectroscopy
TEM	Transmission electron microscopy
SEM	Scanning electron microscopy
